# Comparative outcomes of surpass streamline and evolve flow diverters in intracranial aneurysms: a comprehensive systematic review and meta-analysis of location, size, and morphology

**DOI:** 10.1007/s10143-025-04062-3

**Published:** 2026-01-21

**Authors:** Farhang Rashidi, Mohammad Amin Habibi, Mohammadmahdi Sabahi, Mohammad Reza Arshadi, Mohammad Sina Mirjnani, Parisa Javadnia, Parastoo Radnia, Mohammad Mofatteh, Mohammad Sadegh Mashayekhi, Ehsan Dowlati, Farhan Siddiq, Mubashir Pervez, Kunal Vakharia, Andrew Bauer, Michal Obrzut

**Affiliations:** 1 Department of Neurosurgery, Tehran University of Medical Sciences, Tehran, Iran; 2 School of Medicine, Tehran University of Medical Sciences, Tehran, Iran; 3 Department of Neurological Surgery, Pauline Braathen Neurological Center, Cleveland Clinic Florida, Weston, FL USA; 4 School of Medicine, Dentistry and Biomedical Sciences, Queen’s University Belfast, Belfast, UK; 5 Division of Neurosurgery, Department of Surgery, The Ottawa Hospital, Ottawa, ON Canada; 6 Department of Neurosurgery, North Shore University Hospital, Manhasset, NY USA; 7 Department of Neurological Surgery, University of Missouri School of Medicine, Columbia, MO USA; 8 Department of Neurosurgery and Brain Repair, University of South Florida, Morsani College of Medicine, Tampa, FL USA; 9 Department of Neurosurgery, University of Oklahoma Health Sciences Center, Oklahoma City, OK USA; 10 Section of Neurointerventional Radiology, Cleveland Clinic Florida, 2950 Cleveland Clinic Blvd., Weston, FL 33331 USA

**Keywords:** Aneurysm, Flow diverter, Surpass, Streamline, Evolve

## Abstract

The Surpass flow diverter, including Streamline and Evolve, features a unique design optimized for improved conformability, opening, and wall apposition. Its unique pore structure allows for deployment across a wide range of arterial diameters, contributing to its high occlusion rates. The current study aims to compare the efficacy and safety of the Surpass Streamline and Evolve in treating intracranial aneurysms, with a focus on location, size, and morphology. A systematic review and meta-analysis was carried out according to the PRISMA guidelines, utilizing PubMed, Web of Science, Scopus, and Embase, from inception to August 1st, 2024. Relevant data were extracted, and data synthesis was performed using STATA v.17. The analysis included 27 studies with 1,826 patients (76.7% female; ages 28–84 years) and 2,006 intracranial aneurysms (1,743 anterior, 222 posterior; 28 blister, 20 dissecting, 116 fusiform, and 1,597 saccular). The pooled complete occlusion rate at the final follow-up was 77%, with no significant differences between anterior (80%) and posterior (64%) circulation aneurysms (*P* = 0.15) or small (70%) and large/giant (79%) aneurysms (*P* = 0.36). Morphological analysis revealed that blister, dissecting, saccular, and fusiform aneurysms had 100%, 65%, 55%, and 79% complete occlusion rates, respectively (*P* < 0.001). Surpass Streamline had 81% complete occlusion success versus 69% for Evolve (*P* = 0.04). The overall adequate occlusion rate was 90%, with Streamline at 92% and Evolve at 88% (*P* = 0.35). Higher occlusion rates were observed in posterior aneurysms (100% vs. 92%; *P* < 0.001). Large/giant aneurysms had a higher rate of adequate occlusion (100% vs. 88%; *P* = 0.12). Morphological analysis showed that blister and dissecting aneurysms had 100% adequate occlusion, fusiform aneurysms had 75% adequate occlusion, and saccular aneurysms had 96% adequate occlusion (*P* = 0.04). Mortality, complications and in-stent thrombosis were significantly reduced with Evolve (*P* = 0.03, 0.001 and 0.000, respectively). Our study indicates the efficacy and safety of the Surpass flow diverter for treating intracranial aneurysms, offering a satisfactory occlusion rate. The surpass Evolve was associated with a lower incidence of major complications.

**Supplementary Information:**

The online version contains supplementary material available at 10.1007/s10143-025-04062-3.

## Introduction

Intracranial aneurysms have a prevalence rate of 3.2% in the general population [1]. Although most aneurysms remain asymptomatic, if untreated, they can pose a significant risk of rupture, depending on their size and anatomical location. The treatment landscape for intracranial aneurysms has increasingly shifted toward endovascular approaches, with flow diverters (FD) emerging as a promising technology in aneurysm management [2]. FDs, deployed through minimally invasive endovascular procedures, help to mitigate the risks associated with open microsurgery. FDs also exhibit increased rates of complete occlusion with fewer complications such as recurrence or bleeding, when compared to other endovascular treatments such as stent-assisted coiling [3].

Unlike traditional treatments that provide immediate occlusion, FD therapy depends on complex interactions between the device and the arterial endothelium, as well as the aneurysm’s cellular, molecular, and morphological characteristics [4] [5]. Long-term follow-up studies are vital for confirming successful occlusion and detecting possible complications [6]. Additionally, as with other endoluminal treatments and stents, dual antiplatelet therapy is currently required for all FDs regardless of surface modification. Therefore, their acute use in ruptured aneurysms is controversial.

Recent advancements in FD technology have resulted in the development of improved devices, including the Surpass Streamline and its latest iteration, the Surpass Evolve (Stryker Neurovascular). Recently, the Surpass Elite has also become available although there are currently limited studies regarding this new FD [7]. These devices have been shown enhanced safety and efficacy, with higher rates of complete occlusion and reduced risk of complications in comparison with other FDs. The Surpass Streamline, compared to earlier generations, features a greater number of wires, higher mesh density, and modifications in metal strut design. Specifically, it incorporates a cobalt-chromium stent with 12 platinum wires. These enhancements, result in increased radial force and allow for longer lengths (up to 50 mm in the Evolve model), make the device particularly suitable for treating giant and large aneurysms by ensuring better vessel wall apposition and coverage of the aneurysm neck [9]. The Surpass Evolve builds on the strengths of the Streamline model, maintaining the use of a cobalt-chromium stent with platinum wires but with fewer total wires (64 compared to Streamline’s 72 or 96, depending on diameter). Despite the reduction in wire count, Surpass Evolve’s narrower width and specific design maintain the mesh density and enhance navigability through the microcatheter and tortuous anatomy [10] [11].

Despite these advancements, complications such as intraparenchymal hemorrhage, thromboembolic events, ischemic stroke, and aneurysm rupture remain concerns. Therefore, the objective of this study is to evaluate the safety and efficacy of the Surpass device in treating intracranial aneurysms and to conduct a comprehensive comparison regarding device type and aneurysm nature, including location, size, and morphology.

## Materials and methods

The current systematic review has been previously registered in the International Prospective Register of Systematic Reviews (PROSPERO) receiving the identification code CRD42024509753. The research was conducted following the guidelines of preferred reporting items for systematic reviews and meta-analyses (PRISMA) [12].

### Search strategy

Four different electronic databases, PubMed/Medline, Scopus, Web of Science, and Embase, were reviewed from inception until August 1 st, 2024, and subsequently again, until June 11th, 2025, to ensure up-to-date evidence. No additional filter was applied for study type and publication year. The electronic databases were comprehensively searched by the search strategy provided individually for each database using relative keywords of “Intracranial”, “Aneurysm”, “Flow diverter”, and “Surpass”. The search strategies are provided in detail in Supplementary File 1.

### Eligibility criteria

Inclusion criteria are original English studies on patients diagnosed with intracranial aneurysms, investigating Surpass FDs (regardless of the generation). The exclusion criteria consisted use of (1) stents or other endoluminal devices including Flow Re-Direction Endoluminal Device (FRED) and Pipeline Embolization Device (PED), (2) no reported outcome or complications rates, (3) non-original studies and case reports, (4) series of less than five samples, and (5) non-human subject studies. In cases where studies used the same cohort of patients, the most recent study was chosen. Of note, during our study period there were no studies evaluating the newer Surpass Elite.

### Study selection and data extraction

The data of each electronic database were inserted into EndNote v.21. After eliminating duplicate articles, two independent reviewers examined the articles in a two-step process involving screening titles and abstracts to identify the relevant studies. Subsequently, a full text assessment procedure was undertaken to ascertain that the studies met the required criteria. A third reviewer verified the study selection process. Two other reviewers independently carried out the extraction of data from the included studies and inserted them into a predesigned Excel sheet. Any challenges and conflicts were resolved through group discussion and, if necessary, involvement of a third author.

### Definitions, classifications, and device description

The study employed the Raymond-Roy Occlusion Classification (RROC), Simple Measurement of Aneurysm Residual after Treatment (SMART), and O’Kelly-Marotta grading scale (OKM) to categorize aneurysm occlusion. Complete occlusion was defined as RROC I, OKM D, and SMART 4. Near-total occlusion, or remnant necks, were classified as RROC II, OKM C, and SMART 3. The remaining cases were categorized as residual aneurysms, indicating incomplete occlusion. Additionally, adequate occlusion was defined as RROC I or II, OKM C or D, and SMART 3 or 4.

When classifying aneurysms as anterior or posterior, some included studies considered posterior communicating arteries as anterior [9] [13] [14]. To maintain consistency and ensure more robust data for subgroup analysis, we also applied this classification criterion to other included studies based on Ten Brinck et al. [15]. It should be noted, however, that using the Surpass device in the posterior circulation is off-label.

A study by Ocal et al. [16] investigated two patient groups: one treated solely with the Surpass device and the other with Surpass plus stent implantation. We only included data from the first group to avoid potential confounding effects from the second (stented) group.

### Quality assessment and risk of bias

The Newcastle-Ottawa Scale (NOS) was utilized to assess the quality of the included studies, which includes three domains: selection, comparability, and exposure. Two independent reviewers assessed the quality of the included studies based on the mentioned domains. Conflicts were resolved through discussion with a senior author. Studies were categorized based on obtained scores to 1–3 as low quality, 4–6 as fair quality, and 7–9 as high quality (Supplementary File 2).

### Data synthesis and Meta-analysis

A reasonable measure of effect size was determined in line with the Cochrane Handbook for systematic reviews of interventions. Additionally, the provided pooled assessments were evaluated utilizing a random-effect model with a Restricted Maximum Likelihood (REML). The random-effect model was favored over the fixed-effect model, offering complementary information with a higher 95% confidence interval (CI). Subgroup analysis was performed accordingly, and in the case of three or more subgroups, post-hoc analysis was performed elaborating the details. Leave-one-out analyses and meta-regression were also performed wherever required, along with the small-study effect (Egger’s test) for publication bias. All statistical computations were executed using STATA V.17.

## Results

### Study selection process

**Fig. 1:**
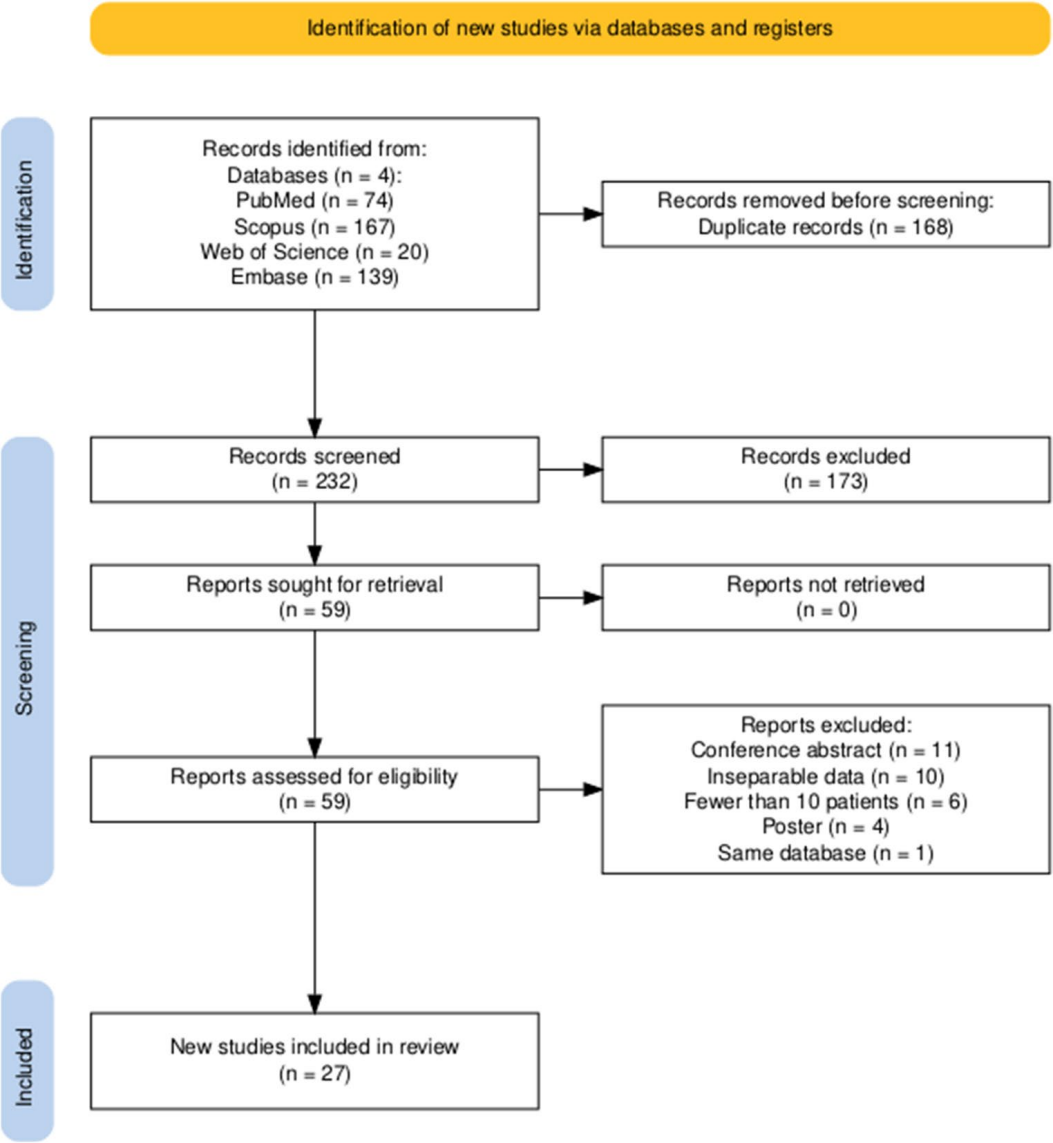
Study selection process

A total of 400 studies were retrieved, and after removing 168 duplicates, 232 titles/abstracts were assessed. Of these, 173 studies were excluded as they were deemed irrelevant to our study. Eventually, 27 studies met the mentioned criteria and were included in the current systematic review and meta-analysis. The PRISMA flow chart in Fig. 1 depicts the details of the study selection process.

Notably, multiple studies with overlapping data were identified. For instance, a relevant study by Meyers et al. [17] reporting the one-year outcome of the SCENT trial was excluded because their second paper, reporting the three-year outcome, had been published. However, three papers [18–20] with similar authors reported outcomes of individuals from the SCENT trial, and we found no specific reason to exclude them despite the possibility of overlap. Additionally, during the revision of the current study, the five-year report of the SCENT trial [21] was published, so we substituted the recent report with the previous one [19].

### Study characteristics

The included studies [9–11] [13–16] [18–20] [22–38] were published between 2013 and 2024 and included two clinical trials. A total of 1,826 patients (76.7% females) and 2,006 aneurysms were recorded. 1,743 anterior and 222 posterior circulation aneurysms were specified. Considering morphology, 28 blister, 20 dissecting, 116 fusiform, and 1,597 saccular aneurysms were recorded. Regarding device type, 17 studies evaluated the Surpass Streamline and 10 studies evaluated the Surpass Evolve. Of the aneurysms treated, 781 (38.9%) were managed using the Surpass Evolve, while 1,225 (61.1%) were treated with the Surpass Streamline (Table 1).

**Table 1:** Study characteristics

Author (Year)	Country	Patients (F)	Age	Aneurysms	Device Type	Location (*n*)	Size (*n*) mm	Morphology (*n*)	Mortality (%)
Meyers et al. (2025) [21]*	Multicenter	180 (165)	61 (9.9)	180	Streamline	Ant (180)	L (167), G (13)	Blist (1), Fusi (15), Sacc (164)	5.0
Dmytriw et al. (2024) [23]	Multicenter	90 (71)	56.9 (12.1)	90	Streamline	Ant (90)	8.9 (6.8)	Sacc (76), Other (14)	NA
Vivanco-Suarez et al. (2024) [22]	USA	305 (256)	59 (50–67)	332	Evolve	Ant (316), Post (16)	S (161), M (109), L (55), G (7)	Blist (7), Diss (5), Fusi (10), Pseudo (1), Sacc (309)	0.7
Gupta et al. (2024) [26]	USA	51 (48)	57.34 (12.27)	80	Evolve	Ant (76), Post (4)	S&M (60), L (10), G (10)	Fusi (2), Pseudo (2), Sacc (76)	0
Bibi et al. (2024) [25]	France	116 (93)	55 (47–63)	120	Evolve	Ant (102), Post (18)	6.6 (4.2)	Blist (1), Fusi (3), Sacc (116)	1.7
Han et al. (2023) [29]	South Korea	40 (28)	59.1	41	Evolve	NA	13.2 (5.53)	NA	0
Vivanco-Suarez et al. (2023) [24]	USA	277 (202)	60 (50–68)	314	Streamline	Ant (279), Post (35)	5.87 (5.52)	Fusi (29), Sacc (267), Other (22)	2.2
Sayin et al. (2023) [11]	Turkey	41 (29)	56.3 (37–78)	52	Evolve	Ant (47), Post (5)	S (48), M (3), L (1)	Diss (5), Fusi (2), Sacc (45)	2.4
Kan et al. (2023) [18]*	USA	38 (34)	63	38	Streamline	Ant (38)	L&G (38)	Sacc (38)	NA
Gupta et al. (2023) [30]	USA	55 (45)	56 (32–83)	69	Streamline	Ant (67), Post (2)	S&M (61), L (8)	Fusi (3), Sacc (66)	3.6
Field et al. (2023) [31]	USA	23 (17)	60.5	23	Evolve	Ant (18), Post (5)	7.1 (3.7)	Sacc (21)	NA
Teranishi et al. (2022) [36]	Japan	26 (24)	(46–86)	26	Streamline	Ant (26)	L&G (26)	Fusi (6), Sacc (20)	NA
Rautio et al. (2022) [28]	Finland	29 (21)	(32–72)	30	Evolve	Ant (24), Post (6)	S&M (20), L&G (10)	Fusi (2), Sacc (28)	6.9
Jee et al. (2022) [32]	South Korea	31 (15)	56.3 (12.2)	31	Evolve	Ant (20), Post (11)	S&M (4), L (17), G (10)	Sacc (11), Other (20)	3.2
Feigen et al. (2022) [33]	USA	45 (35)	56.8 (14.0)	45	Streamline	Ant (44), Post (1)	4.95 (3.49)	Fusi (1), Sacc (44)	0
Achey et al. (2022) [9]	USA	36 (25)	58 (46–63)	42	Streamline	Ant (38), Post (4)	S&M (28), L (13), G (1)	Blist (2), Fusi (1), Sacc (39)	8.3
Siddiqui et al. (2022) [27]	USA	11	NA	11	Streamline	Post (11)	L&G (11)	Fusi (11)	0
Ten Brinck et al. (2021) [15]	Netherlands	19 (15)	56.4 (9.9)	21	Streamline	Ant (21)	S&M (8), Other (13)	NA	0
Maus et al. (2021) [37]	Germany	42 (32)	58 (28–84)	46	Evolve	Ant (41), Post (5)	S&M (34), L&G (12)	Blist (4), Diss (2), Fusi (10), Sacc (30)	2.4
Orru et al. (2020) [10]	Canada	25 (20)	(36–86)	26	Evolve	Ant (25), Post (1)	S (15), L (8), G (2)	Diss (1), Fusi (1), Sacc (21)	0
Ocal et al. (2019) [16]	Turkey	35 (21)	49 (15)	41	Streamline	Ant (34), Post (7)	9.1	Fusi (9), Sacc (32)	NA
Topcuoglu et al. (2018) [34]	Turkey	24 (16)	52.3 (19.7)	25	Streamline	Ant (21), Post (4)	15.7 (8.8)	NA	0
Mahajan et al. (2018) [38]	USA	16 (8)	(35–74)	16	Streamline	Ant (13), Post (3)	S (13), M (2), L (1)	Blist (5), Fusi (1), Sacc (6), Fusisacc (4)	6.3
Taschner et al. (2016) [35]	Germany	53 (21)	(16–79)	52	Streamline	Post (52)	S (4), M (13), L&G (34)	Sacc (12), Other (40)	17.3
Colby et al. (2015) [20]*	USA	20 (18)	63.3 (1.3)	20	Streamline	Ant (20)	L (20)	Fusi (7), Sacc (13)	0
Wakhloo et al. (2014) [13]	Netherlands	161 (116)	(28–82)	186	Streamline	Ant (159). Post (27)	S (53), M (64), L&G (69)	Blist (7), Sacc (125), Other (54)	4.3
Vries et al. (2013) [14]	Netherlands	37 (26)	(32–79)	49	Streamline	Ant (44), Post (5)	NA	Blist (1), Diss (7), Fusi (3), Sacc (38)	0

Regarding aneurysm size: small (< 5 mm), medium (5–10 mm), large (10–25 mm), and giant (> 25 mm)

*Blist* Blister, *Diss* Dissecting, *Fusi* Fusiform, *Pseudo* Pseudoaneurysm, *Sacc* Saccular, *Ant* Anterior, *Post* Posterior, *S* Small, *M,* Medium, *L* Large, *G* Giant

*Possible data overlap may be present between the maked studies

### Complete occlusion rate

**Fig. 2:**
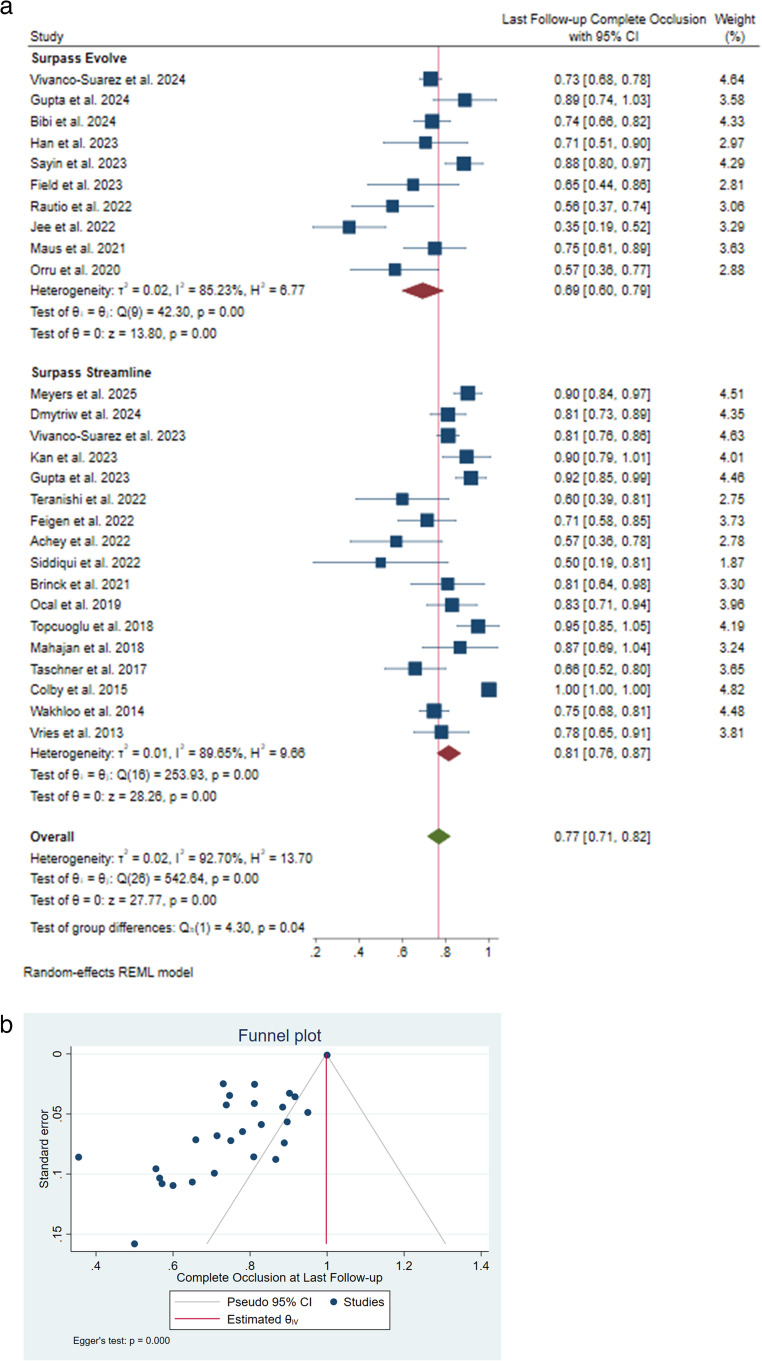
(**a**) Complete occlusion rate as last follow-up forest plot. (**b**) Complete occlusion rate as last follow-up funnel plot

The complete occlusion rate was reported in 6–[9], 12 − [9–31], 24–[26], 36 − [18], and 60-month [21] follow-up durations, and the pooled rate at these time points were 55% [95% CI: 0.43–0.66], 75% [95% CI: 0.66–0.84], 89% [95% CI: 0.74–1.03], 83% [95% CI: 0.71–0.95], and 90% [95% CI: 0.84–0.97], respectively. Altogether, the complete occlusion rate at all studies’ last follow-up was 77% [95% CI: 0.71–0.82]. Surpass Streamline and Surpass Evolve had a complete occlusion pooled rate of 81% [95% CI: 0.78–0.87; I²=89.65%, 𝜏²=0.01] and 69% [95% CI: 0.60–0.79; I²=85.23%, 𝜏²=0.02], respectively. The difference between these two groups was significant (I²=92.70%, 𝜏²=0.02; *P* = 0.04) (Fig. 2A). Significant heterogeneity was reported; however, no outliers were identified in the sensitivity analysis, and the meta-regression analysis revealed that the source of heterogeneity was not the publication year or follow-up duration. Significant publication bias was also reported (*P* < 0.001) (Fig. 2B).

### Complete occlusion based on location

A subgroup analysis of aneurysm location based on circulation (anterior and posterior) was conducted in 17 studies [9–11] [13–15] [18–20] [22] [23] [27] [28] [35–38] that reported separate data regarding each circulation. The pooled complete occlusion rate at the last follow-up was 80% [95% CI: 0.73–0.86] and 64% [95% CI: 0.43–0.85] in the anterior and posterior circulation, respectively. The difference between the two groups was not significant (I²=99.98%, 𝜏²=0.05; *P* = 0.15).

### Complete occlusion based on size

A subgroup analysis of aneurysm size (< 10 mm and > 10 mm) was conducted in 12 studies [9–11] [18–20] [22] [27–29] [36] [38], which reported separate data based on aneurysm size. The pooled complete occlusion rate at the last follow-up was 70% [95% CI: 0.55–0.85] and 79% [95% CI: 0.68–0.90] in small/medium and large/giant aneurysms, respectively. The difference between the two groups was not significant (I²=99.97%, 𝜏²=0.03; *P* = 0.36).

### Complete occlusion based on morphology

A subgroup analysis of aneurysm morphology (blister, dissecting, fusiform, and saccular) was conducted in 11 studies [9–11] [14] [18] [20] [27] [28] [36–38], which reported separate data according to each morphology. The pooled rate of complete occlusion at the last follow-up was 100% [95% CI: 1.00–1.00], 65% [95% CI: 0.17–1.14], 55% [95% CI: 0.28–0.81], and 79% [95% CI: 0.68–0.89] in blister, dissecting, fusiform, and saccular aneurysms, respectively. The difference between these four groups was significant (I²=100.00%, 𝜏²=0.11; *P* < 0.001). However, based on post-hoc analysis, no groups were statistically different from each other (*P* = 0.23). These controversial results could be explained by the probable high variability and small effect size of the studies.

### Adequate occlusion rate

**Fig. 3:**
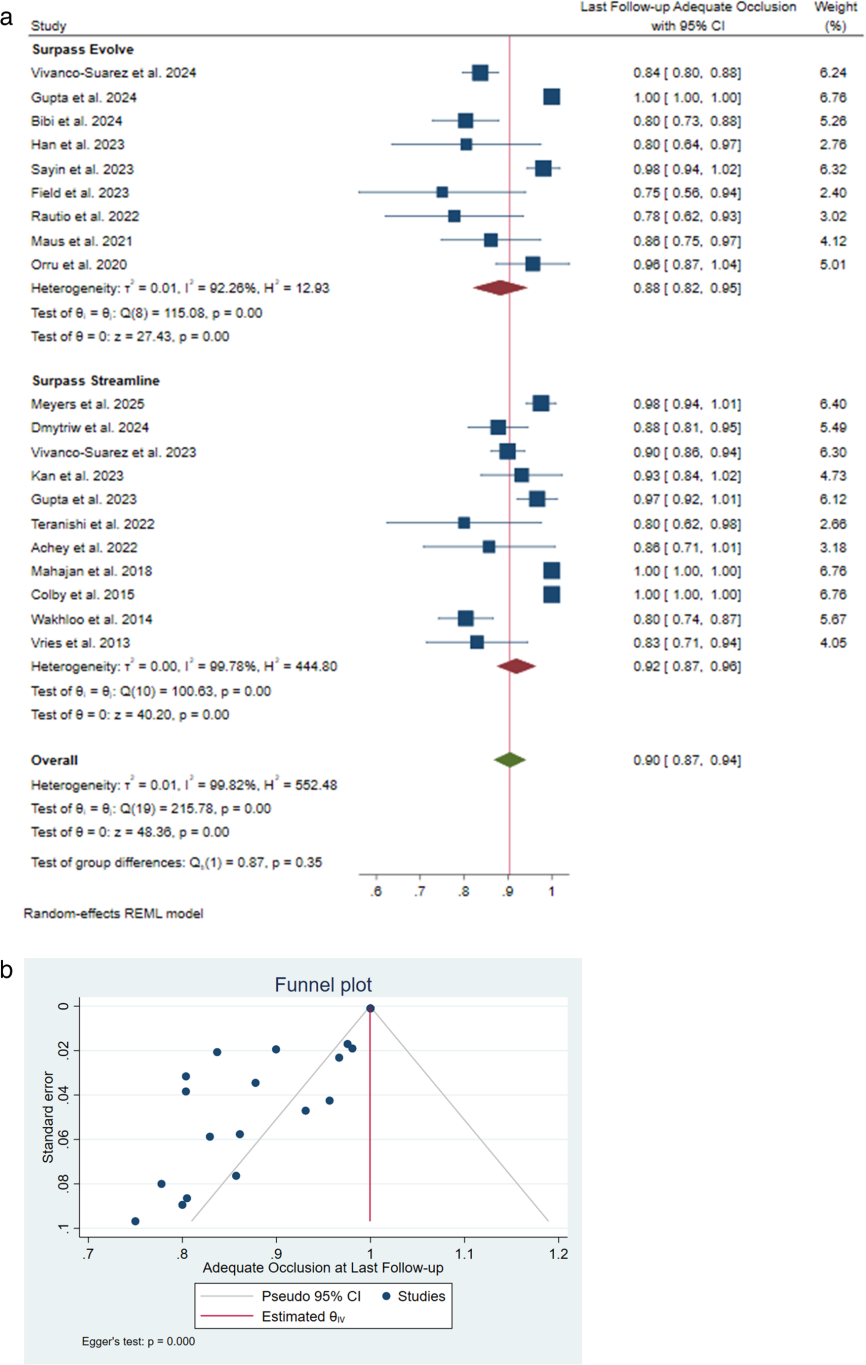
(**a**) Adequate occlusion rate at last follow-up forest plot. (**b**) Adequate occlusion rate at last follow-up funnel plot

An adequate occlusion rate was reported in 6–[9], 12 − [9], 24–[26], and 36 − [18], and 60-month [21] follow-up durations, and the pooled rates were 81% [95% CI: 0.71–0.91], 88% [95% CI: 0.80–0.95], 100% [95% CI: 1.00–1.00], 98% [95% CI: 0.93–1.03], and 98% [95% CI: 0.94–1.01], respectively. Altogether, an adequate occlusion rate at all studies’ last follow-up was 90% [95% CI: 0.87–0.94]. Surpass Streamline and Surpass Evolve had an adequate occlusion pooled rate of 92% [95% CI: 0.87–0.96; I²=99.78%, 𝜏²=0.00] and 88% [95% CI: 0.82–0.95; I²=92.26%, 𝜏²=0.01], respectively. The difference between these two groups was not significant (I²=99.82%, 𝜏²=0.01; *P* = 0.35) (Fig. 3A). Significant heterogeneity was reported; however, no outliers were identified in the sensitivity analysis, and the source of heterogeneity was not the publication year or follow-up duration according to the meta-regression. Significant publication bias was also reported (*P* < 0.001) (Fig. 3B).

### Adequate occlusion based on location

A subgroup analysis based on circulation (anterior and posterior) was conducted in 13 studies [9–11] [13] [14] [18–20] [23] [28] [36–38] that reported separate data regarding each circulation. The pooled rate of adequate occlusion at the last follow-up was 92% [95% CI: 0.87–0.96] and 100% [95% CI: 1.00–1.00] in the anterior and posterior circulation, respectively. Although utilizing the Surpass device in posterior circulation is off-label, the adequate occlusion rate was significantly better in the posterior than the anterior circulation, probably due to a limited included study, which yielded a high but imprecise occlusion rate (I²=99.92%, 𝜏²=0.00; *P* < 0.001).

### Adequate occlusion based on size

A subgroup analysis of aneurysm size (< 10 mm and > 10 mm) was conducted in 10 studies [9–11] [18–20] [28] [29] [36] [38] that reported separate data according to aneurysm size. The pooled rate of adequate occlusion at the last follow-up was 88% [95% CI: 0.72–1.03] and 100% [95% CI: 1.00–1.00] in small/medium and large/giant aneurysms, respectively. The difference between the two groups was not significant (I²=99.91%, 𝜏²=0.00; *P* = 0.12).

### Adequate occlusion based on morphology

A subgroup analysis of aneurysm morphology (blister, dissecting, fusiform, and saccular) was conducted in 10 studies [9–11] [14] [18] [20] [28] [36–38], which reported separate data in terms of each morphology. The pooled rate of adequate occlusion at the last follow-up was 100% [95% CI: 1.00–1.00], 100% [95% CI: 1.00–1.00], 75% [95% CI: 0.50–1.01], and 96% [95% CI: 0.93–1.00.93.00] in blister, dissecting, fusiform, and saccular aneurysms, respectively. The difference between these four groups was significant (I²=100.00%, 𝜏²=0.05; *P* = 0.04). However, based on post-hoc analysis, no groups were statistically different from each other (*P* = 0.25). These controversial results could be due to the high variability and small effect size of the included studies.

### Major complications

Major complications included events such as ischemic or hemorrhagic strokes, subarachnoid hemorrhages, carotid-cavernous fistulas, aneurysm ruptures, or any other permanent morbidity that did not result in death.

**Fig. 4:**
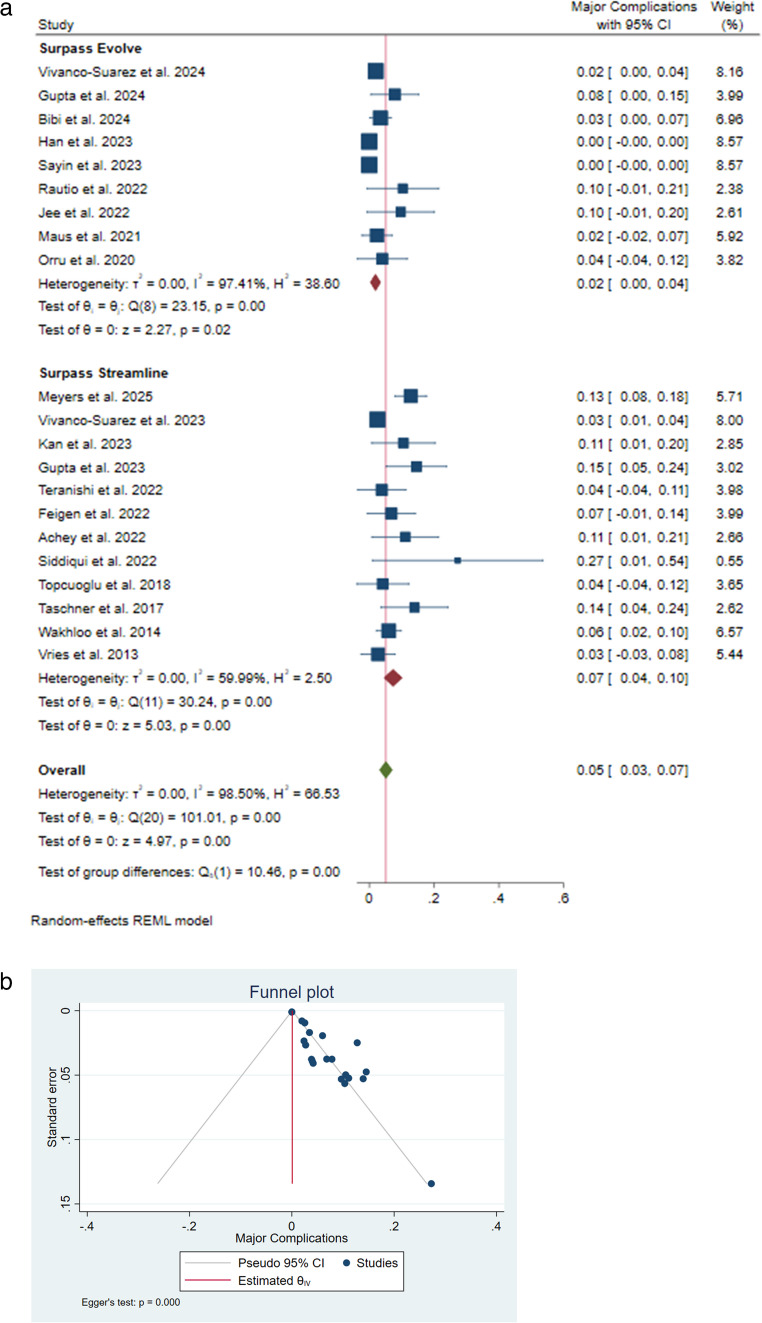
(**a**) Complications rate forest plot. (**b**) Complications rate funnel plot

The pooled incidence rate of major complication was 5% [95% CI: 0.03–0.07], reported in a total of 21 studies [9–11] [13] [14] [18] [19] [22] [24–30] [32–37]. In the subgroup analysis, the pooled major complication rate with the use of the Surpass Streamline and Evolve was 7% [95% CI: 0.04–0.10] and 2% [95% CI: 0.00–0.04.00.04], respectively, indicating a significantly lower rate with Surpass Evolve (I²=98.50%, 𝜏²=0.00; *P* < 0.001) (Fig. 4A). Publication bias was reported to be significant (*P* < 0.001) (Fig. 4B).

### Other adverse events

Pooled retreatment rates were 3% [95% CI: 0.01–0.04] reported in 10 studies [13] [15] [19] [22–24] [27] [29] [34] [37]. The pooled parent artery/in-stent stenosis rate was 6% [95% CI: 0.02–0.10] reported in 14 studies [11] [13] [19] [22] [24–26] [28–31] [34] [36] [37]. In-stent thrombosis was reported in 11 studies [10] [11] [13] [24] [26] [28–30] [32] [37] [38] with a pooled rate of 3% [95% CI: 0.01–0.04]. In all three analyses, there was an insignificant difference between Surpass Streamline and Surpass Evolve, except for in-stent thrombosis, with Surpass Evolve exhibiting a significantly lower rate (*P* < 0.001).

### Mortality

**Fig. 5:**
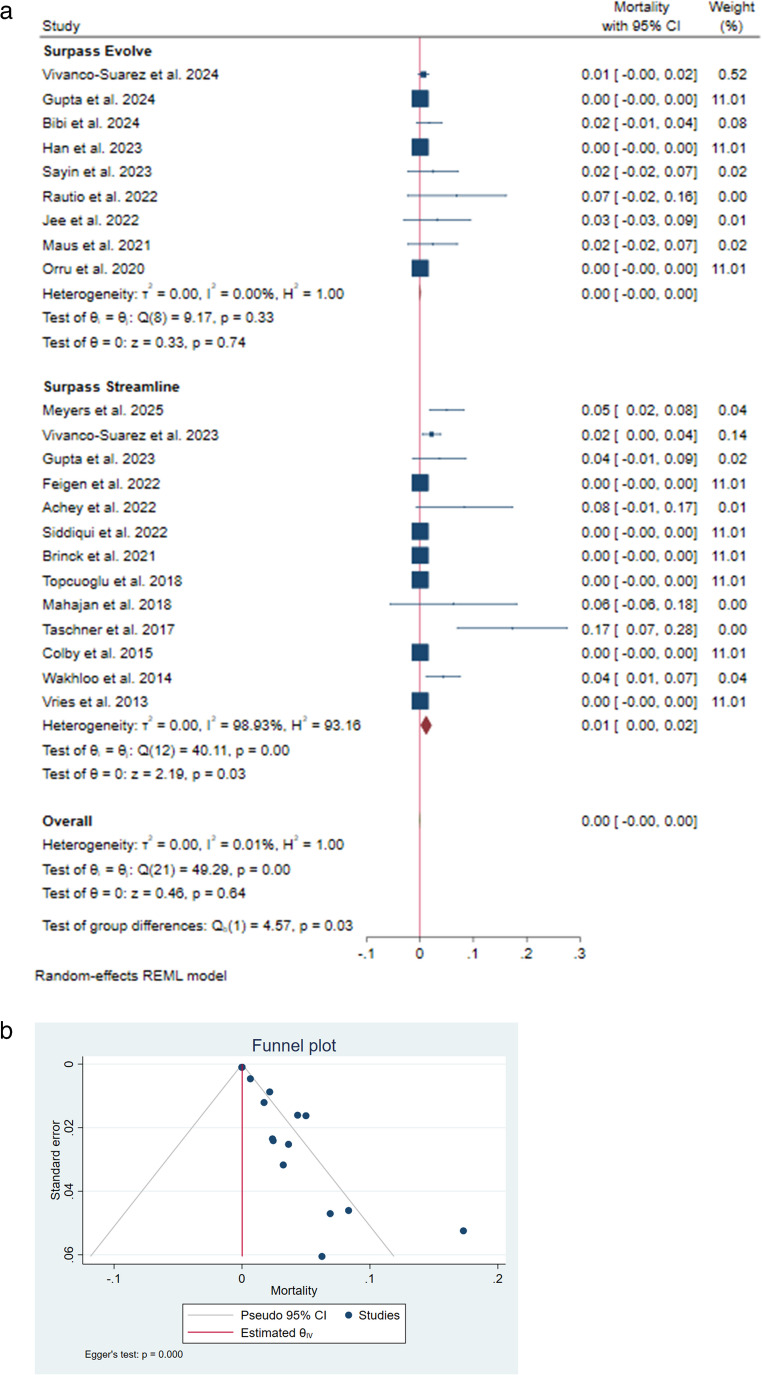
(**a**) Mortality rate forest plot. (**b**) Mortality rate funnel plot

All-cause mortality was reported in 22 studies [9–11] [13–15] [19] [20] [22] [24–30] [32–35] [37] [38], ranging from 0% to 17%, with a pooled rate of < 1% [95% CI: close to zero, indicating minimal variation]. In the subgroup analysis, mortality rates of Surpass Streamline and Evolve were calculated as 1% [95% CI: 0.00–0.02.00.02] and 0% [95% CI: close to zero, indicating minimal variation], respectively. The difference between the two groups was significant (I²=00.01%, 𝜏²=0.00; *P* = 0.03) (Fig. 5A). Publication bias was also significant (*P* < 0.001) (Fig. 5B).

### Publication bias and quality assessment

All of the included studies were assessed using the NOS. Accordingly, almost all included studies were considered to have high quality (Supplementary File 2). In terms of publication bias, funnel plots are presented in Supplementary File 3.

## Discussion

The current systematic review and meta-analysis is the most updated study investigating Surpass devices. Unlike previous meta-analyses, such as Issa et al. [39], which aggregated occlusion and complication rates without generation-specific comparisons; Florez-Perdomo et al. [40], which involved a smaller cohort and lacked stratification by device iteration or detailed aneurysm subgroups; Rodriguez-Calienes et al. [41], which analyzed Surpass Evolve outcomes with limited subgroup granularity; and the recent Jazayeri et al. study [42] that focused on Evolve safety and efficacy without direct comparison to Streamline, our research offers a distinct head-to-head comparison of Streamline and Evolve. Additionally, it systematically stratifies by aneurysm location, size, and morphology, standardizes occlusion definitions, and includes long-term trajectory data. Previous reviews provided data on overall occlusion rates and complication incidences but failed to specify whether design modifications in Evolve lead to measurable safety improvements or efficacy trade-offs across specific aneurysm subtypes. This study demonstrates that Evolve results in significantly lower rates of thrombosis and major complications while maintaining comparable overall occlusion rates. On the other hand, Streamline exhibited a numerically higher complete occlusion rate despite a higher complication rate, which implies a possible efficacy-safety trade-off. Additionally, it reveals nuanced patterns, such as differential occlusion across morphologies, and improved adequate occlusion in posterior and large or giant aneurysms, thereby addressing a critical knowledge gap.

Our analysis revealed pooled complete and adequate occlusion rates of 76% and 90%, respectively, at the last follow-up. Notably, studies with extended follow-up periods showed increasing rates of complete occlusion over time. For instance, Vivanco-Suarez et al. [24] reported an 8.4% increase in complete occlusion at a follow-up period of greater than 12 when compared to an initial follow-up period of 6–12 months. Similarly, a 3-year SCENT study affirmed an 11.1% increase in patients’ total occlusion rates at the end of the third year compared with assessments after the first year (increasing from 66.7% to 77.8%) [19]. Adequate occlusion rates were even higher, likely due to the gradual progression of the endothelialization process, which can vary based on the aneurysm’s anatomical characteristics [4] [5] [43]. Additionally, this increasing rate could be due to patients’ loss during the follow-ups. As for the SCENT trial, the number of patients with radiographical data decreased from 117 to 82 in the third and fifth years of follow-up, resulting in higher proportions of occlusion [21].

While some studies did not document major complications, our analysis indicates that they cannot be entirely disregarded, with a pooled complication rate of 5%. In fact, five studies reported a complication rate exceeding 10% [9]. The most frequent complications were symptomatic ischemic infarctions, often preceded by hemorrhages, typically in the form of subarachnoid hemorrhage. Thromboembolic events, a key complication, can occur due to the thrombogenicity of the device, difficult anatomy increasing procedure time, vessel atherosclerotic disease/stenoses, and aneurysm complexity [44].

As stated earlier, the findings suggest a potential trade-off between efficacy (defined as complete occlusion) and safety (including major complications and thrombosis). Device selection should be individualized based on specific clinical circumstances. In cases where complete occlusion is critical, such as large-neck aneurysms with low hemorrhagic risk and favorable anatomy, Streamline may be a suitable option. Conversely, in situations with heightened thromboembolic or hemorrhagic risks, such as tortuous anatomy or the need for lower device thrombogenicity, Evolve may provide a safety benefit. Due to variations in follow-up durations and case mix among studies, these signals require careful interpretation. Prospective head-to-head trials are necessary to determine if the observed efficacy-safety gradient remains consistent after adjusting for confounding factors.

Likewise, underlying neurological or systemic deficits, such as coagulopathies or other conditions affecting vascular integrity, can lead to severe adverse consequences. In such cases, proper antiplatelet regimens are critical to minimizing thromboembolic events and ensuring the success of the primary therapeutic intervention [45] [46]. Gupta et al. reported a high stroke rate despite appropriate antiplatelet therapy, highlighting the need for cautious selection of treatment modalities based on aneurysm characteristics [30]. They suggested that selecting Surpass Evolve over Surpass Streamline for treating aneurysms in the posterior circulation is preferable due to its lower structural profile, maintainance of mesh density, and lower risk for infarction [30]. Our study is the first to demonstrate the clinical superiority of the Surpass Evolve in reducing major complications; however, further comparative studies are necessary to elucidate the underlying mechanisms and evaluate differences in complete and adequate occlusion rates.

While anterior circulation aneurysms are more common than aneurysms in the posterior circulation, the latter are linked to a greater risk of rupture and subsequent complications [47] [48]. Basilar tip aneurysms pose the highest risk of rupture, followed by those in the posterior and anterior communicating arteries [1]. In our meta-analysis, the rate of adequate occlusion was significantly higher in the posterior than in the anterior circulation. These results, however, may be due to a small sample size and selection bias.

It is worth noting that Surpass Elite represents Stryker’s next-generation product, having received FDA approval and CE Mark clearance in 2024. Its application is specified for adults with unruptured large or giant wide-neck saccular aneurysms (neck ≥ 4 mm or dome-to-neck < 2) or fusiform aneurysms located from the petrous segment to the terminus; safety for ruptured aneurysms remains unverified. In vitro and computational flow dynamics studies demonstrate decreased thrombin generation compared to unmodified comparators, along with favorable flow-diversion characteristics in relation to PED-Shield [7] [8]. Ongoing research aims to assess the safety and efficacy in the context of intracranial aneurysms.

## Strengths and limitations

Even though this is the first comprehensive study investigating the different aspects of the safety and efficacy of the surpass flow diverter, it has some limitations. Many studies incorporated in this meta-analysis were retrospective cohorts, leading to possible selection bias. The use of different imaging techniques, a diverse patient population, and the administration of various types and doses of antiplatelet therapy, combined with the absence of a specific follow-up schedule, all contributed to these limitations. Additionally, subgroup analyses were conducted using the available data, which was limited in some cases, potentially compromising the robustness of the results. For instance, there was no individual data on antiplatelet regimen or ruptured status, which could be potential confounders of the outcome. It is worth mentioning that pooled mortality, while seemingly near zero, has been reported as non-zero in several studies. Our analysis incorporated zero-event studies with continuity corrections, and sensitivity analyses utilizing Generalized linear mixed models (GLMMs) indicated that the pooled rate is below 1%, though it is not precisely zero. The advent of newer generation devices such as Surpass Elite add new opportunity for improvement and research in this arena. Further research, especially randomized clinical trials, could be conducted to further enlighten the efficacy and possible complications of Surpass flow diverters and specify the results according to location, size, aneurysm morphology, vessel anatomy, and device type.

## Conclusion

The Surpass flow diverters, particularly the Surpass Evolve, have demonstrated promising occlusion rates with low rates of major complications and mortality, offering reasonable efficacy and safety for treating intracranial aneurysms. The Surpass Evolve suggests similar occlusion rates with a better safety profile than its predecessor, the Surpass Streamline.

## Supplementary Information

Below is the link to the electronic supplementary material.

Supplementary Material 1 (DOCX 18.5 KB)

Supplementary Material 2 (DOCX 20.8 KB)

Supplementary Material 3 (DOCX 73.6 MB)

## Data Availability

The authors confirm that the data supporting the findings of this study are available within the article.
