# Supplementary material for: Comparative outcomes of surpass streamline and evolve flow diverters in intracranial aneurysms: a comprehensive systematic review and meta-analysis of location, size, and morphology
Source: Neurosurg Rev. 2026 Jan 21;49(1):139. doi: 10.1007/s10143-025-04062-3 (PMC12823732; doi:10.1007/s10143-025-04062-3)
Supplement: Supplementary file 1 — Supplementary Material 1 (DOCX 18.5 KB) [file 10143_2025_4062_MOESM1_ESM.docx]

| **Table S1:** Search strategies for different databases. | | |
| --- | --- | --- |
| Database | Search strategy | Number |
| PubMed | #1 "Intracranial Aneurysm"[Mesh] OR "intracranial aneurysm"[tiab] OR "brain aneurysm"[tiab] OR "cerebral aneurysm"[tiab] OR "cerebral artery aneurysm"[tiab] OR "middle cerebral artery aneurysm"[tiab] OR "cerebral aneurysm*"[tiab] OR "brain aneurysm*"[tiab] OR "Intracranial aneurysm*"[tiab]  #2 “surpass”[tiab] OR “surpass flow diverter”[tiab] OR “surpass evolve flow diverter”[tiab] OR “surpass evolve”[tiab] OR “surpass streamline”[tiab]  #3 #1 AND #2 | **74** |
| Embase | #1 'intracranial aneurysm'/exp OR 'brain artery aneurysm'/exp OR 'arteria cerebri media aneurysm':ti,ab OR 'brain aneurysm':ti,ab OR 'brain vessel aneurysm':ti,ab OR 'cerebral aneurysm':ti,ab OR 'cerebral artery aneurysm':ti,ab OR 'medial brain artery aneurysm':ti,ab OR 'middle brain artery aneurysm':ti,ab OR 'middle cerebral artery aneurysm':ti,ab OR 'cerebral aneurysm*':ti,ab OR 'brain aneurysm*':ti,ab OR 'Intracranial aneurysm*':ti,ab  #2 'surpass'/exp OR 'surpass flow diverter'/exp OR 'surpass evolve flow diverter':ti,ab OR 'surpass evolve':ti,ab OR 'surpass':ti,ab OR 'surpass streamline':ti,ab  #3 #1 AND #2 | **139** |
| Web Of Science | #1 TI= ("surpass" OR "surpass flow diverter" OR "surpass evolve flow diverter" OR "surpass evolve" OR "surpass streamline ")  #2 TI= ("intracranial aneurysm" OR "brain artery aneurysm" OR "arteria cerebri media aneurysm” OR “brain aneurysm” OR “brain vessel aneurysm” OR “cerebral aneurysm” OR “cerebral artery aneurysm” OR “medial brain artery aneurysm” OR “middle brain artery aneurysm” OR “middle cerebral artery aneurysm” OR “cerebral aneurysm*” OR “brain aneurysm*” OR “Intracranial aneurysm*”)  #3 #1 AND #2 | **20** |
| Scopus | #1 TITLE-ABS-KEY (“surpass" OR "surpass flow diverter" OR "surpass evolve flow diverter" OR "surpass evolve" OR "surpass streamline")  #2 TITLE-ABS-KEY (“intracranial aneurysm” OR “brain artery aneurysm” OR “arteria cerebri media aneurysm” OR “brain aneurysm” OR “brain vessel aneurysm” OR “cerebral aneurysm” OR “cerebral artery aneurysm” OR “medial brain artery aneurysm” OR “middle brain artery aneurysm” OR “middle cerebral artery aneurysm” OR “cerebral aneurysm*” OR “brain aneurysm*” OR “Intracranial aneurysm*”)  #3 #1 AND #2 | **167** |

Mesh: Medical Subject Headings.

1 August, 2024

Total: 400

Duplications: 168

For screen: 232
