# Supplementary material for: Comparative outcomes of surpass streamline and evolve flow diverters in intracranial aneurysms: a comprehensive systematic review and meta-analysis of location, size, and morphology
Source: Neurosurg Rev. 2026 Jan 21;49(1):139. doi: 10.1007/s10143-025-04062-3 (PMC12823732; doi:10.1007/s10143-025-04062-3)
Supplement: Supplementary file 2 — Supplementary Material 2 (DOCX 20.8 KB) [file 10143_2025_4062_MOESM2_ESM.docx]

**Supplementary Table 2.** Quality assessment of included studies using Newcastle Ottawa Scale (NOS).

| Author, Year | **Selection** | | | | **Comparability** | | **Outcome/Exposure** | | | **Total (out of 9)** |
| --- | --- | --- | --- | --- | --- | --- | --- | --- | --- | --- |
|  | Representativeness | Selection of non-exposed | Ascertainment of exposure | Demonstration of absence of the outcome of interest | Adjust for the most important risk factors | Adjust for the other risk factors | Assessment of outcome | Follow-up length | Adequacy of follow-up |  |
| Meyers et al. 2025 | * |  | * | * | * |  | * | * | * | 7 |
| Dmytriw et al. 2024 | * | * | * | * | * |  | * | * | * | 8 |
| Vivanco-Suarez et al. 2024 | * |  | * | * | * | * | * | * | * | 8 |
| Gupta et al. 2024 | * |  | * | * | * |  | * | * | * | 7 |
| Bibi et al. 2024 | * |  | * | * | * |  | * | * | * | 7 |
| Han et al. 2023 | * |  | * | * | * |  | * | * | * | 7 |
| Vivanco-Suarez et al. 2023 | * |  | * | * | * |  | * | * | * | 7 |
| Sayin et al. 2023 | * |  | * | * | * |  | * | * | * | 7 |
| Kan et al. 2023 | * |  | * | * | * |  | * | * | * | 7 |
| Gupta et al. 2023 | * |  | * | * | * |  | * | * | * | 7 |
| Field et al. 2023 | * | * | * | * | * |  | * | * | * | 8 |
| Teranishi et al. 2022 | * |  | * | * | * |  | * | * | * | 7 |
| Rautio et al. 2022 | * |  | * | * | * |  | * | * | * | 7 |
| Jee et al. 2022 | * |  | * | * | * |  | * | * | * | 7 |
| Feigen et al. 2022 | * | * | * | * | * | * | * | * | * | 9 |
| Achey et al. 2022 | * |  | * | * | * |  | * | * | * | 7 |
| Siddiqui et al. 2022 | * | * | * | * | * |  | * | * | * | 8 |
| Brinck et al. 2021 | * |  | * | * | * |  | * | * | * | 7 |
| Maus et al. 2021 | * |  | * | * | * |  | * | * | * | 7 |
| Orru et al. 2020 | * |  | * | * | * | * | * | * | * | 8 |
| Ocal et al. 2019 | * |  | * | * | * |  | * | * | * | 7 |
| Topcuoglu et al. 2018 | * |  | * | * | * |  | * | * | * | 7 |
| Mahajan et al. 2018 | * |  | * | * | * |  | * | * | * | 7 |
| Taschner et al. 2017 | * |  | * | * | * |  | * | * | * | 7 |
| Colby et al. 2015 | * |  | * | * | * |  | * | * | * | 7 |
| Wakhloo et al. 2014 | * |  | * | * | * |  | * | * | * | 7 |
| Vries et al. 2013 | * |  | * | * | * |  | * | * | * | 7 |
