# Supplementary material for: Comparative outcomes of surpass streamline and evolve flow diverters in intracranial aneurysms: a comprehensive systematic review and meta-analysis of location, size, and morphology
Source: Neurosurg Rev. 2026 Jan 21;49(1):139. doi: 10.1007/s10143-025-04062-3 (PMC12823732; doi:10.1007/s10143-025-04062-3)
Supplement: Supplementary file 3 — Supplementary Material 3 (DOCX 73.6 MB) [file 10143_2025_4062_MOESM3_ESM.docx]

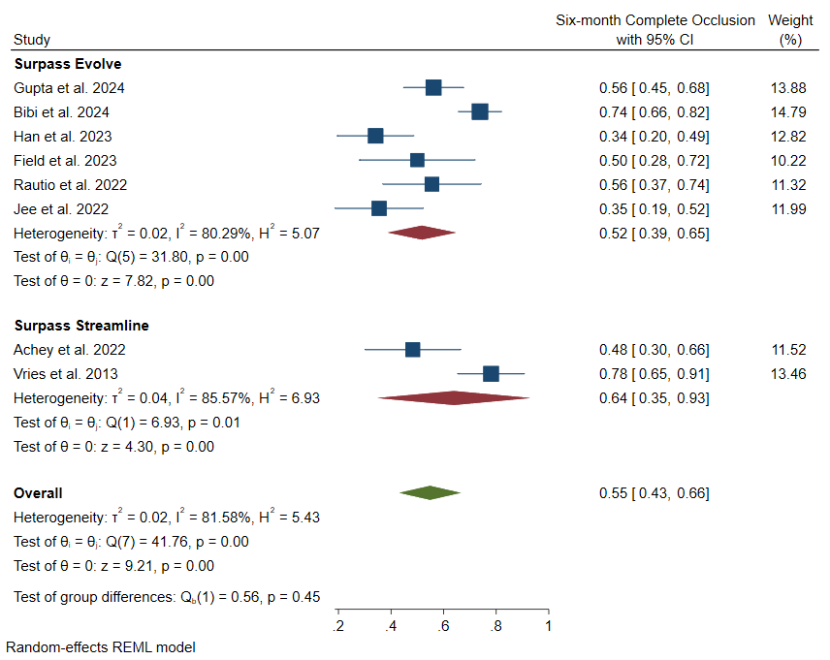


Figure 1 Six-month complete occlusion


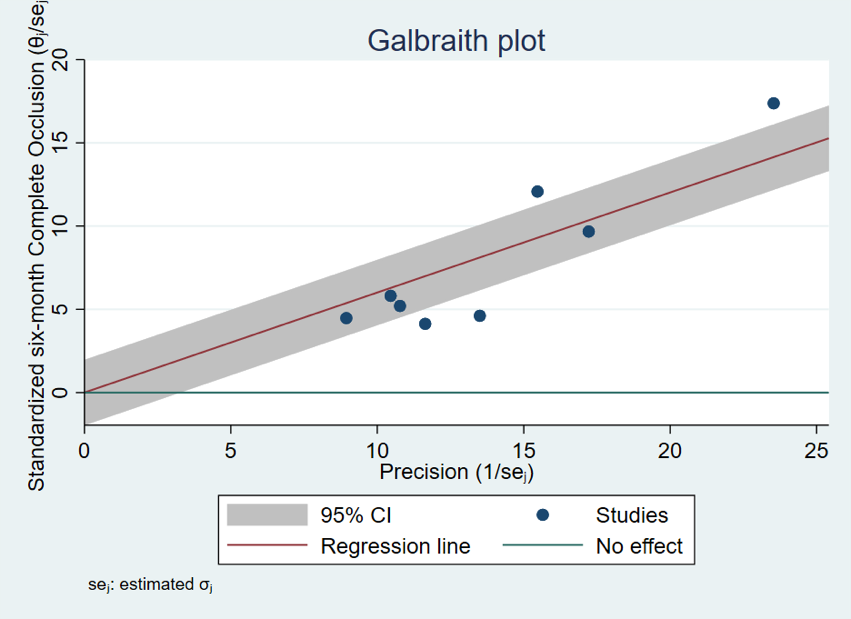


Figure 2 Six-month complete occlusion heterogeneity


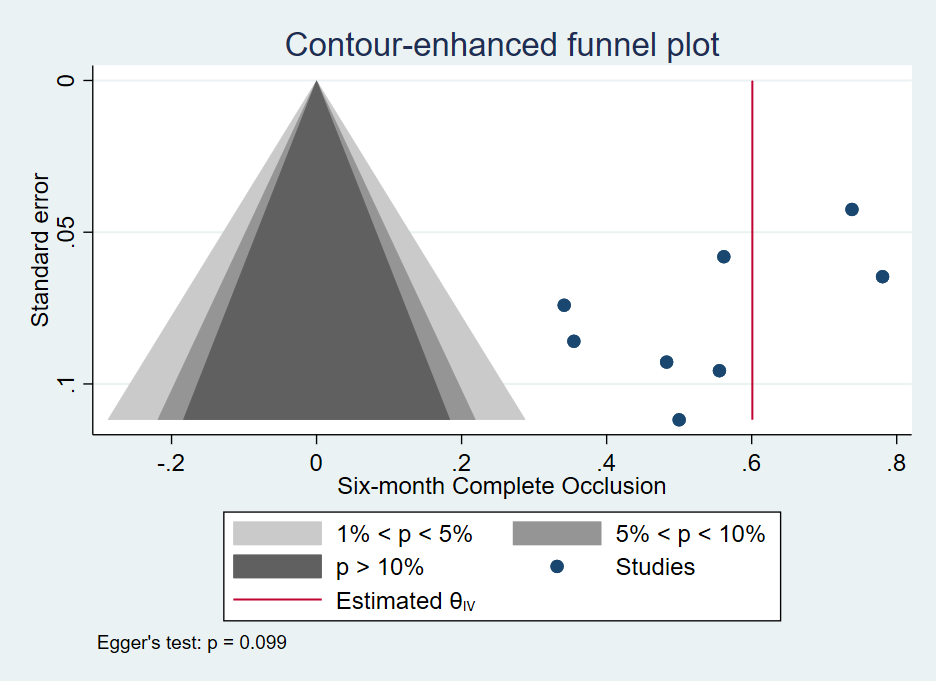


Figure 3 Six-month complete occlusion publication bias


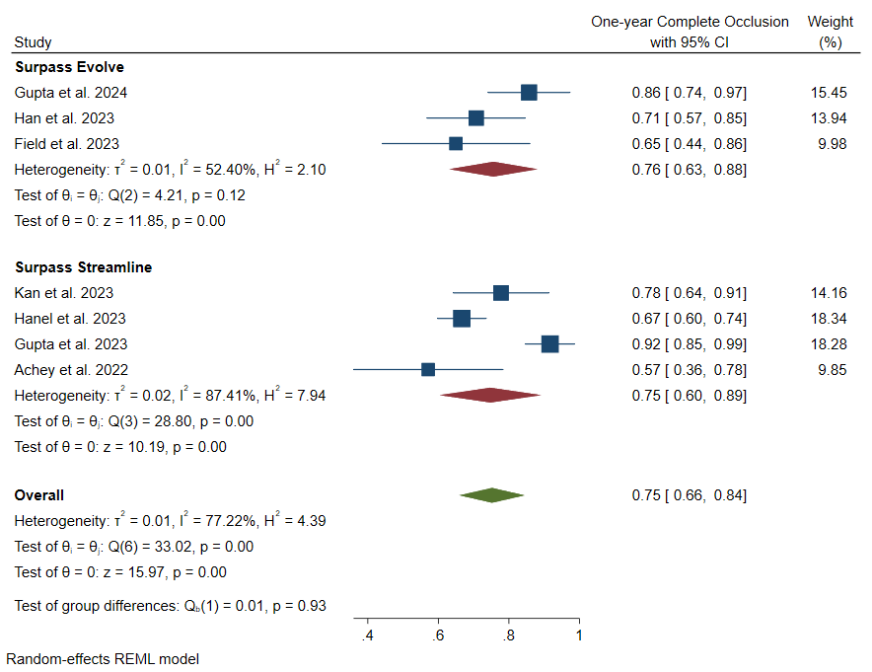


Figure 4 One-year complete occlusion


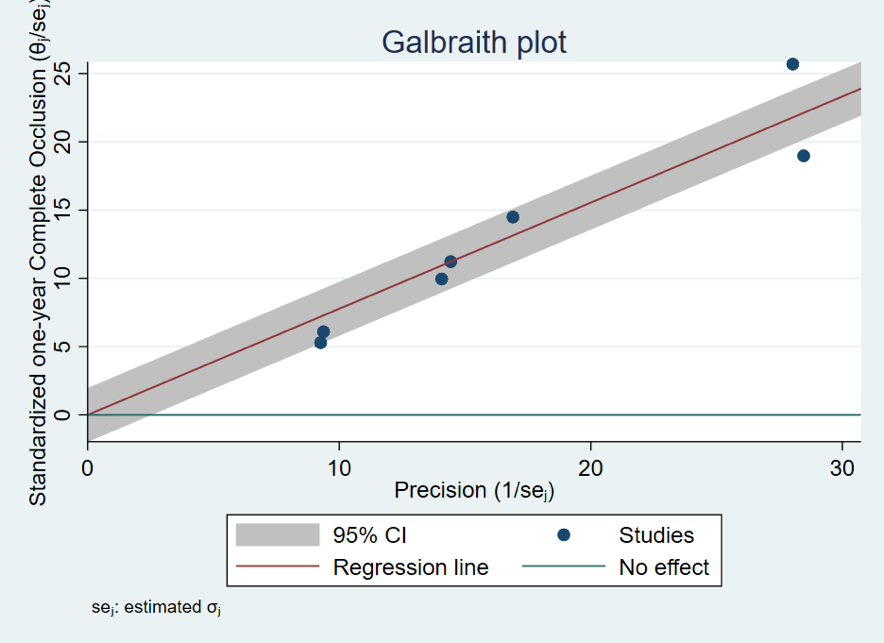


Figure 5 One-year complete occlusion heterogeneity


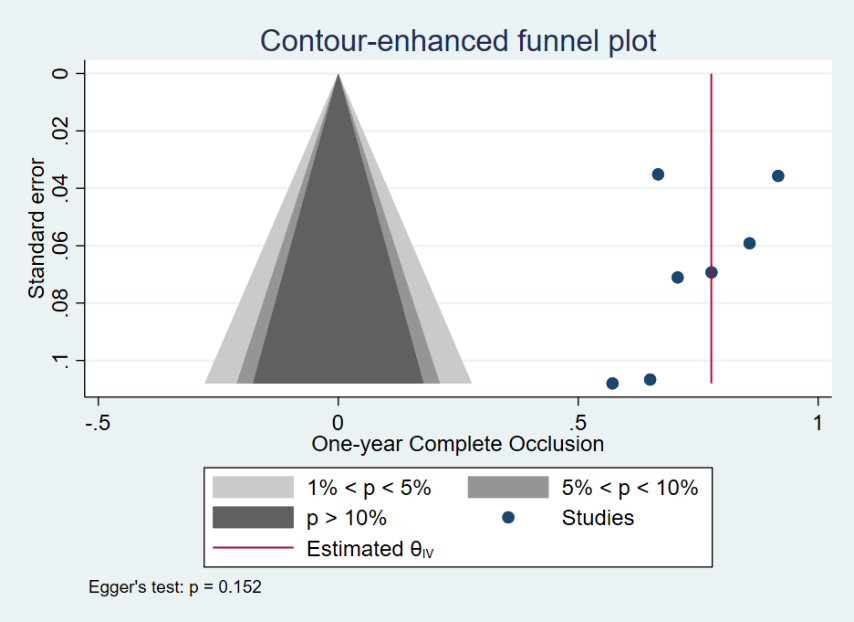


Figure 6 One-year complete occlusion publication bias


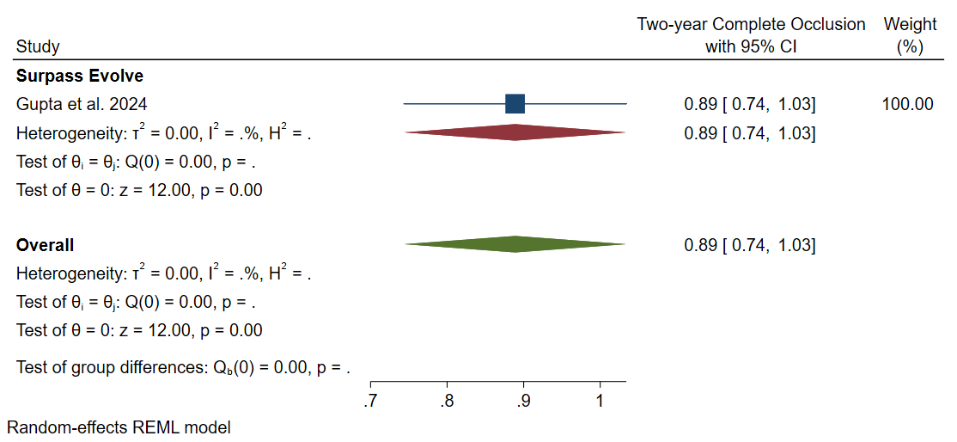


Figure 7 Two-year complete occlusion


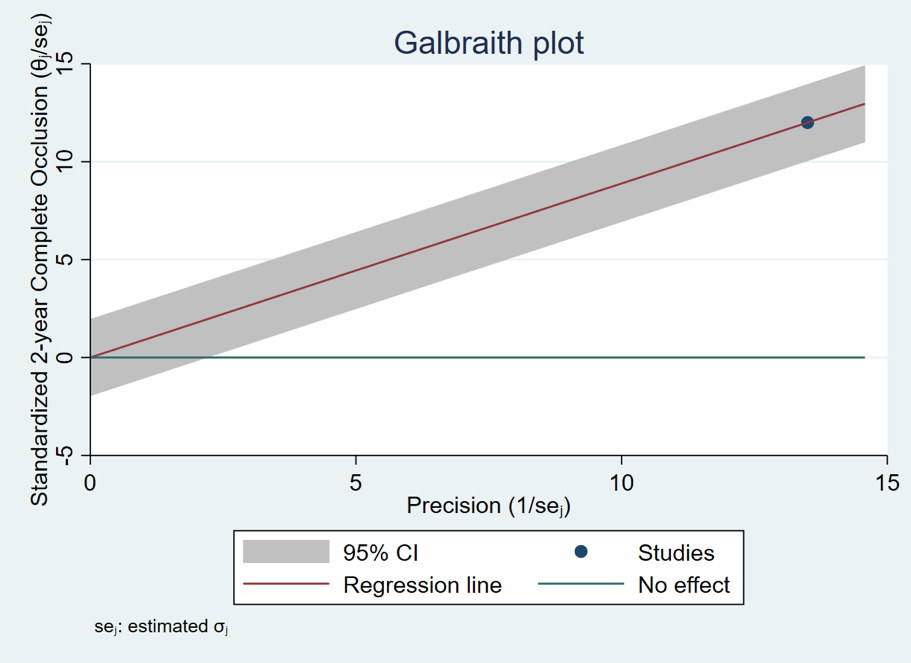


Figure 8 Two-year complete occlusion heterogeneity


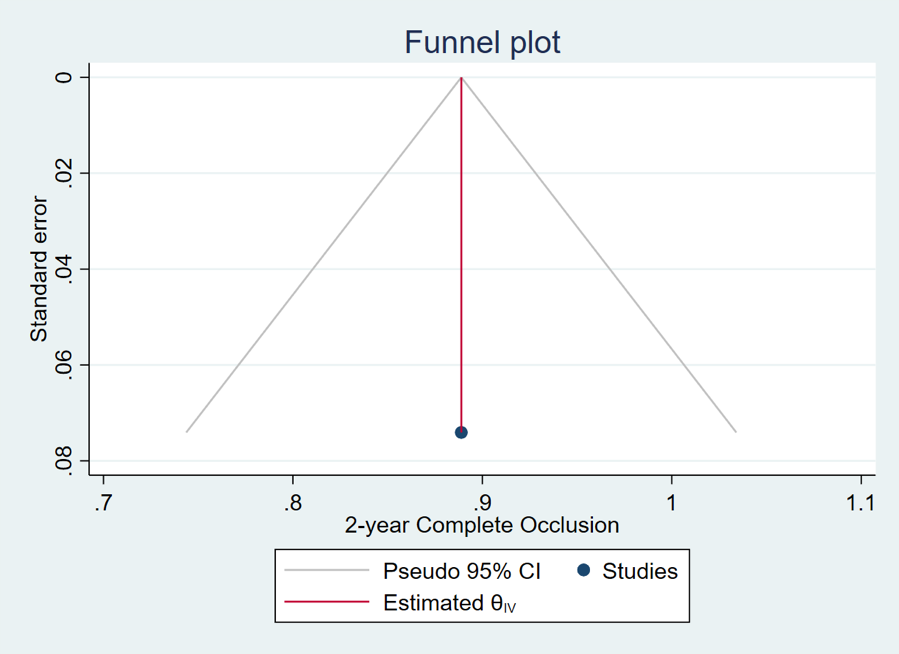


Figure 9 Two-year complete occlusion publication bias


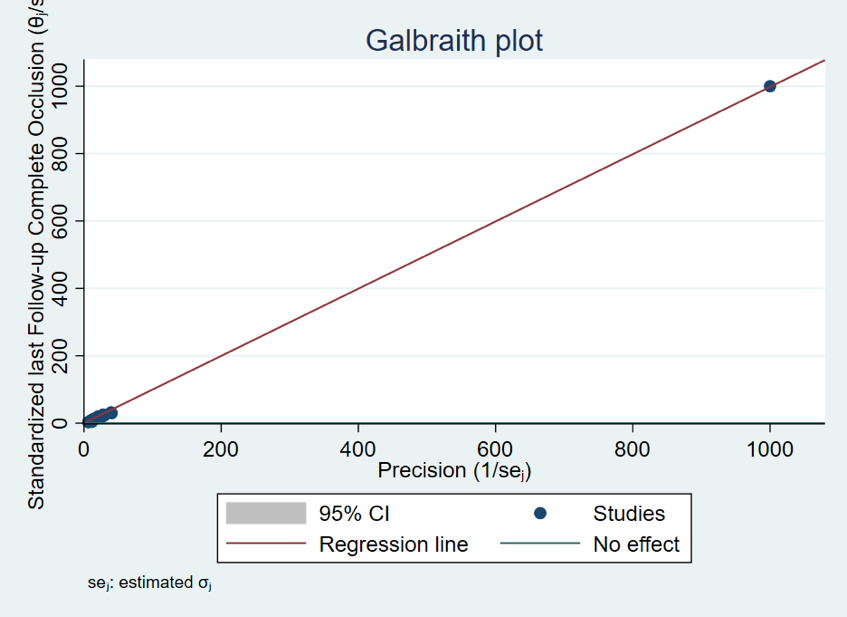


Figure 13 Last follow-up complete occlusion heterogeneity


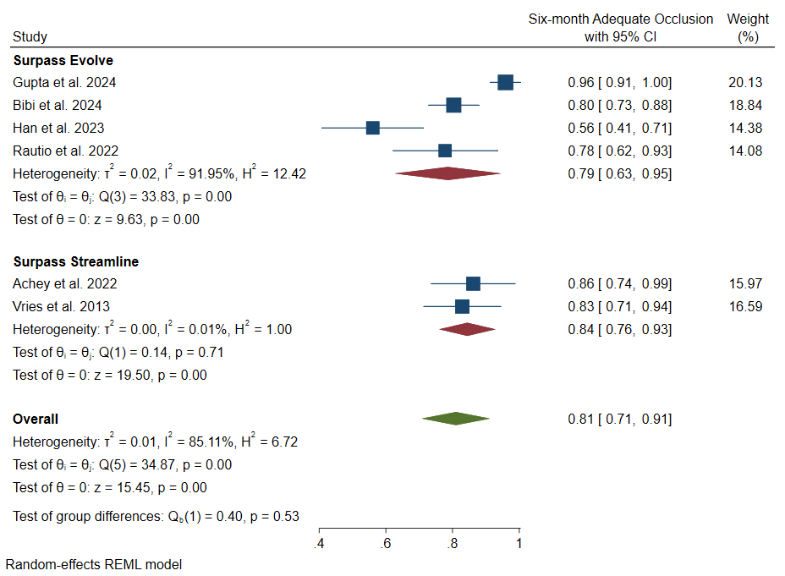


Figure 15 Six-month adequate occlusion


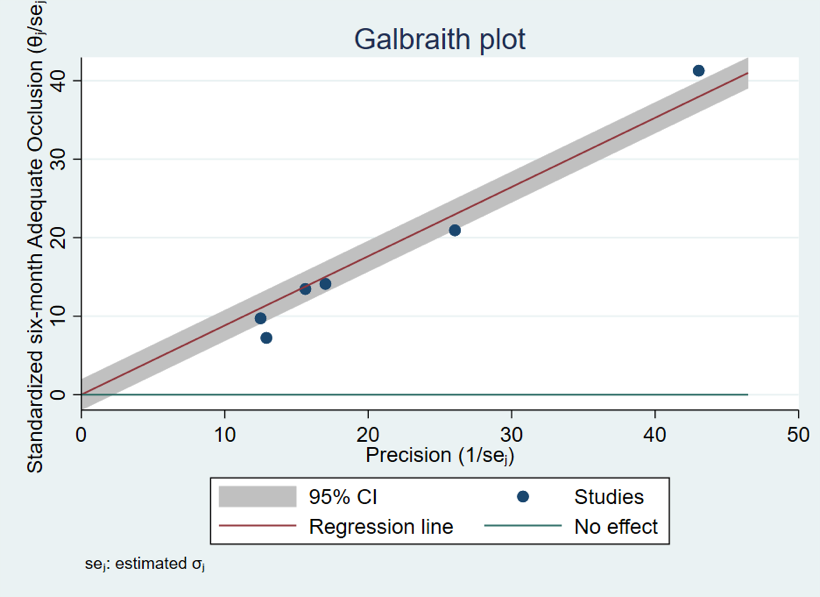


Figure 16 Six-month adequate occlusion heterogeneity


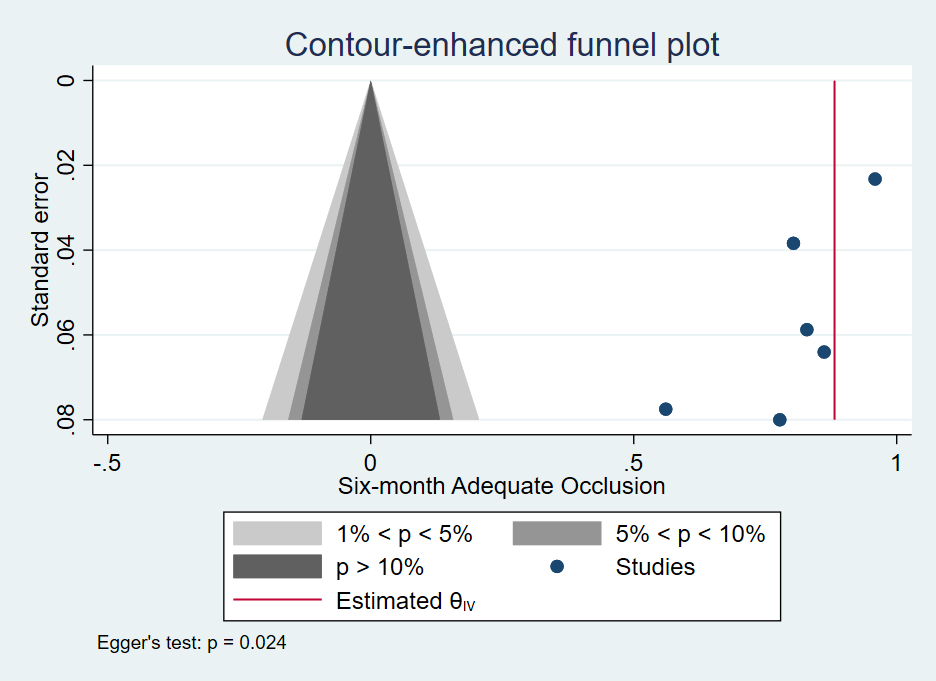


Figure 17 Six-month adequate occlusion publication bias


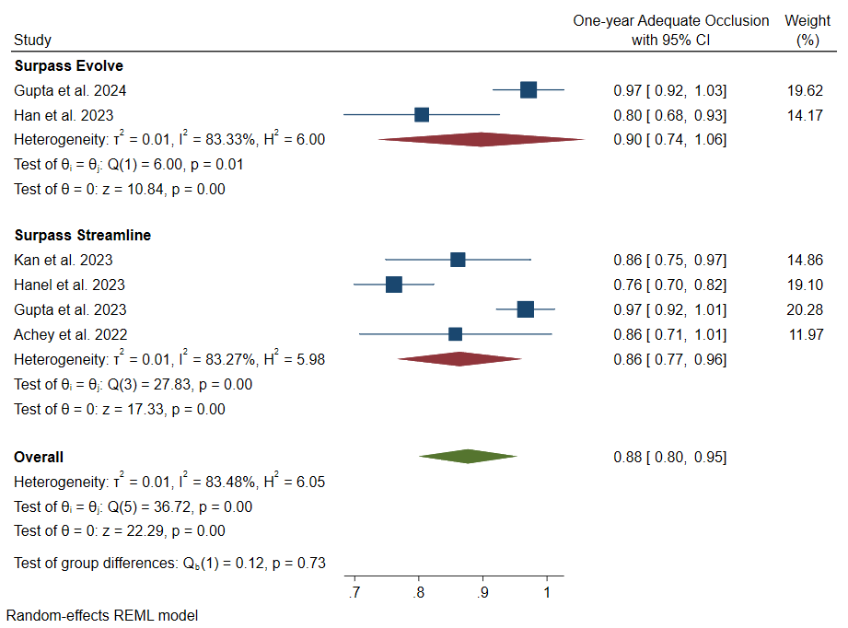


Figure 18 One-year adequate occlusion


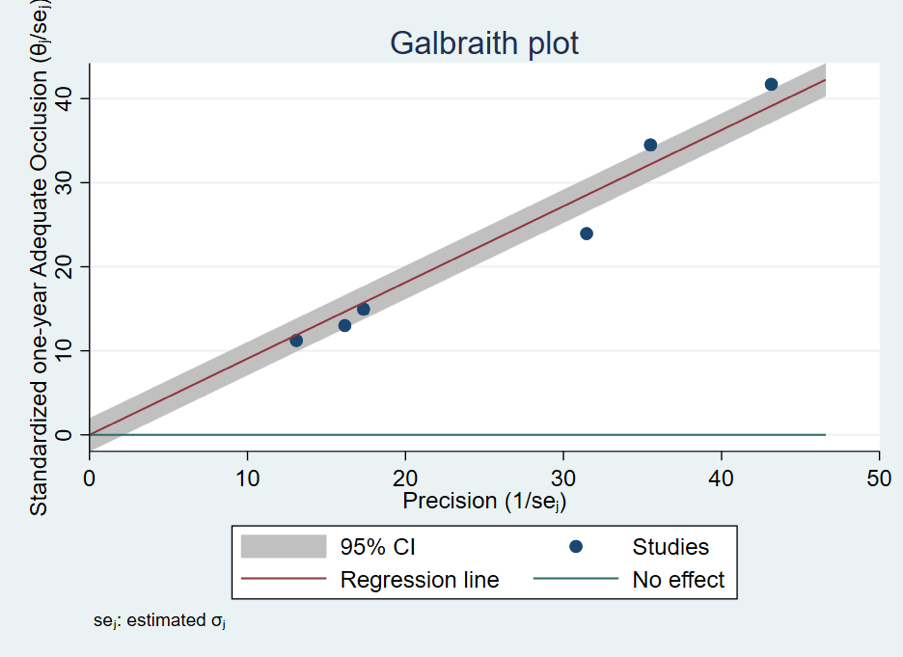


Figure 19 One-year adequate occlusion heterogeneity


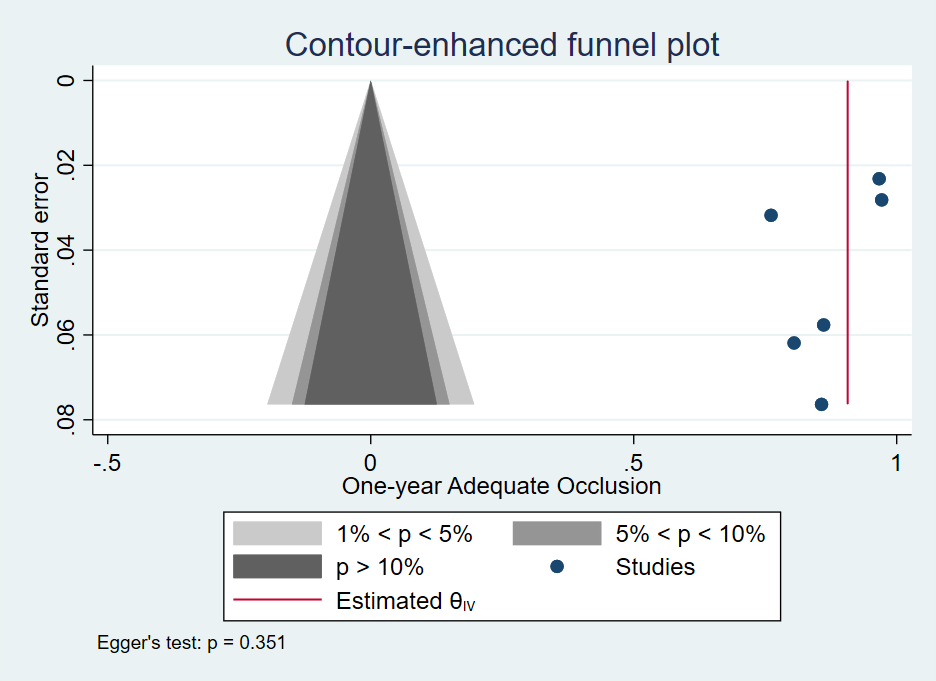


Figure 20 One-year adequate occlusion publication bias


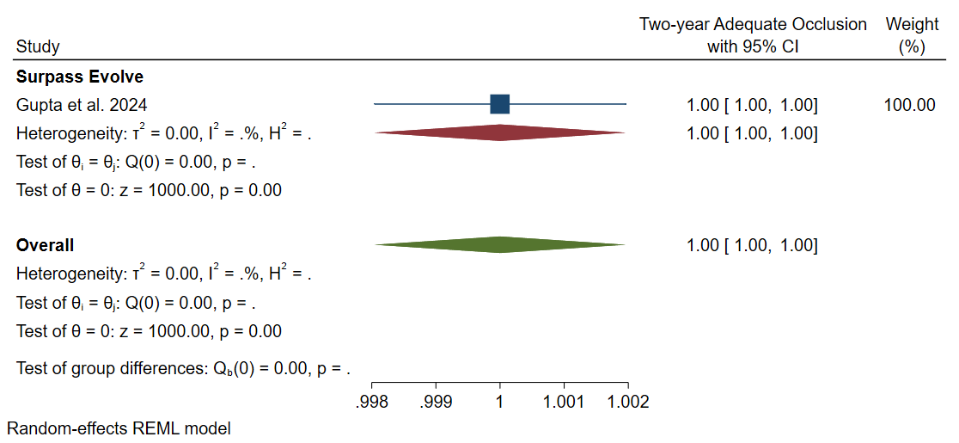


Figure 21 Two-year adequate occlusion


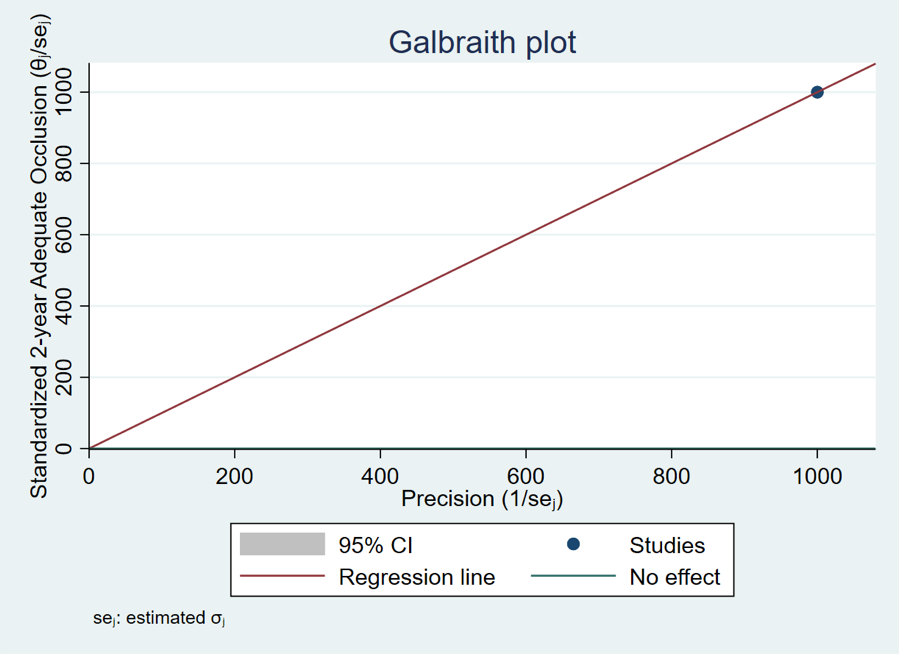


Figure 22 Two-year adequate occlusion heterogeneity


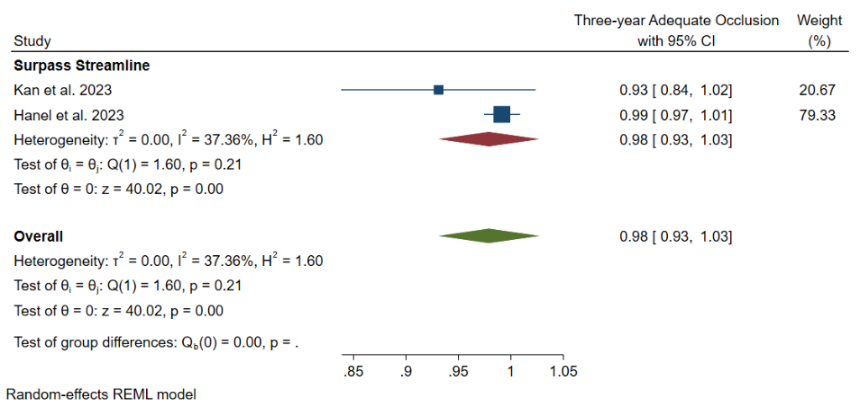


Figure 24 Three-year adequate occlusion


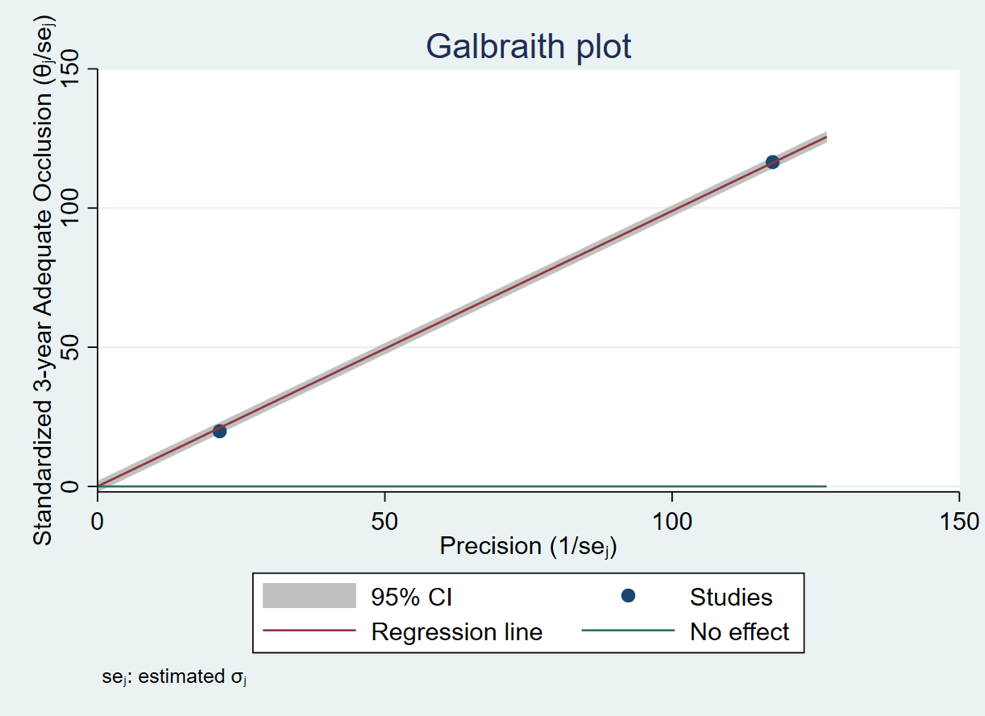


Figure 25 Three-year adequate occlusion heterogeneity


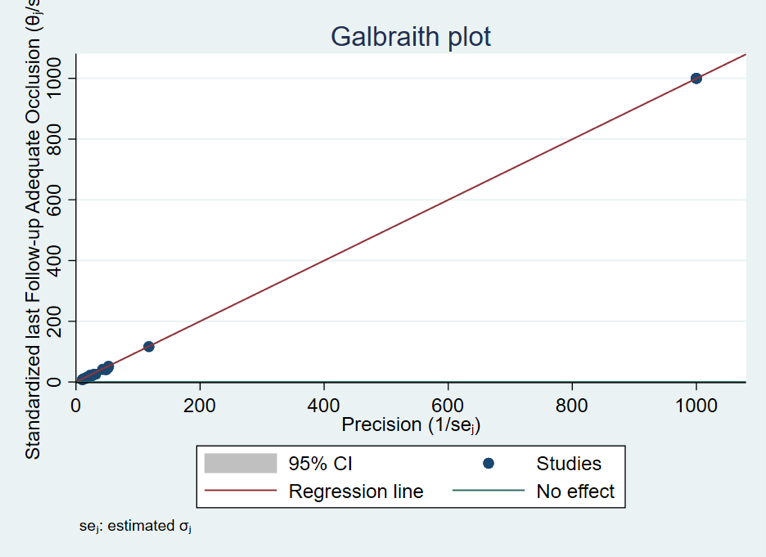


Figure 27 Last follow-up adequate occlusion heterogeneity


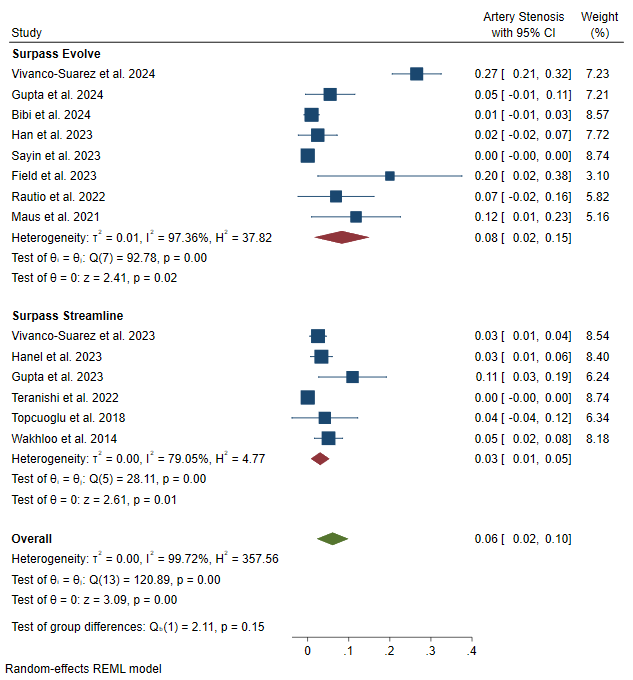


Figure 29 Artery stenosis


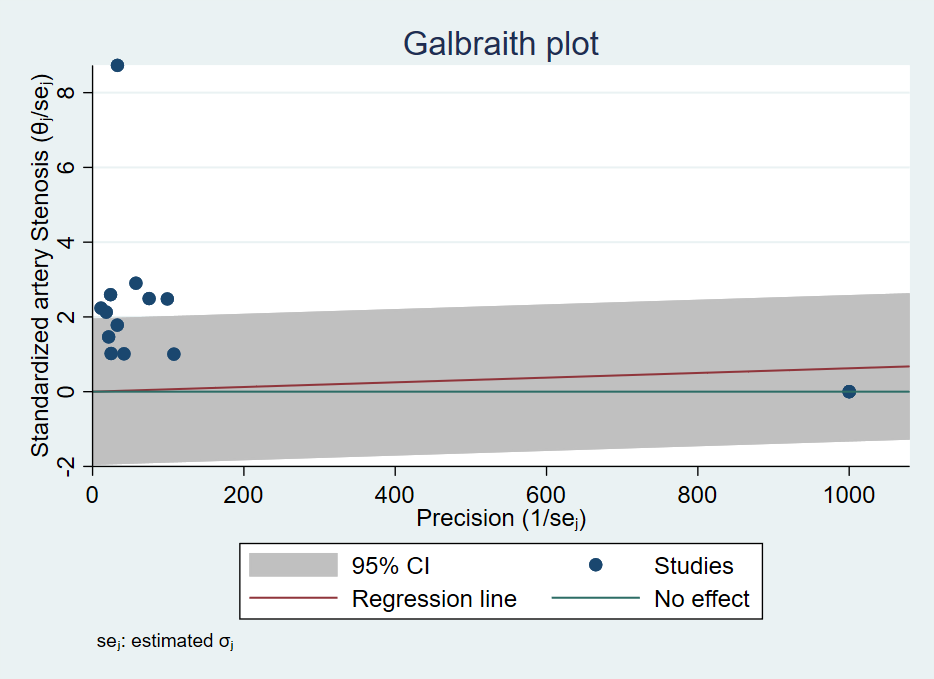


Figure 30 Artery stenosis heterogeneity


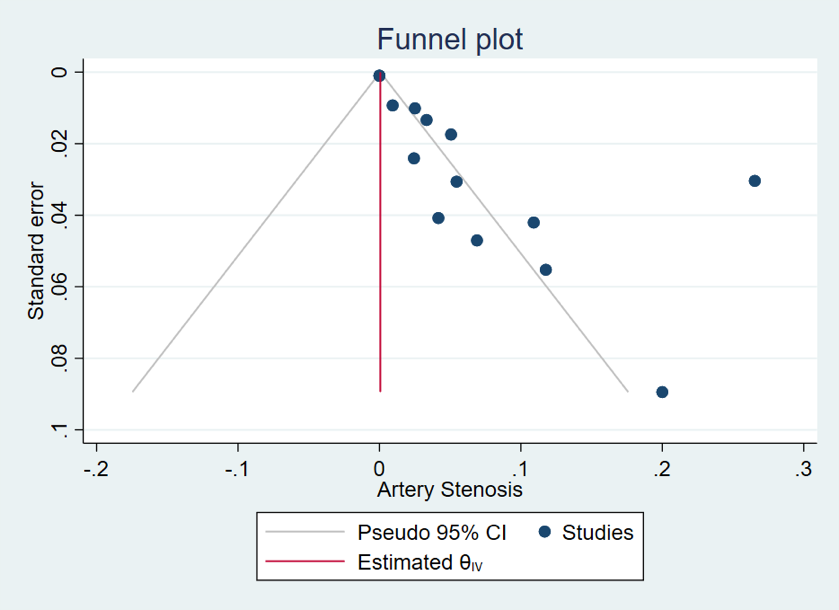


Figure 31 Artery stenosis publication bias


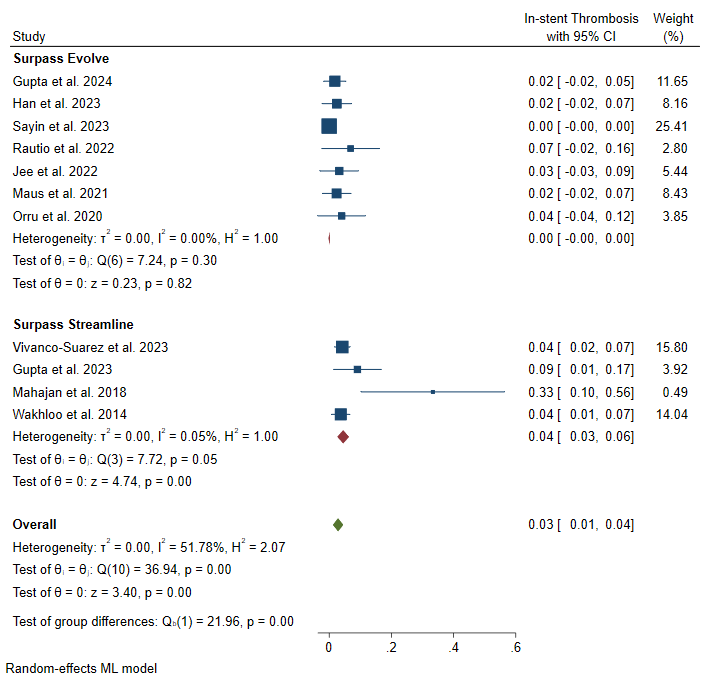


Figure 32 In-stent thrombosis


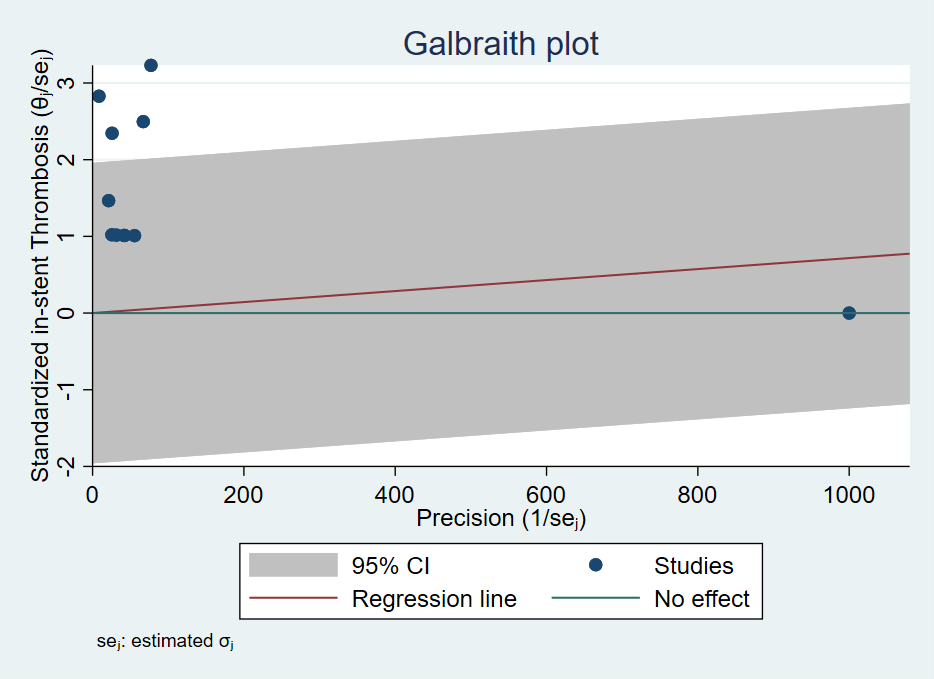


Figure 33 In-stent thrombosis heterogeneity


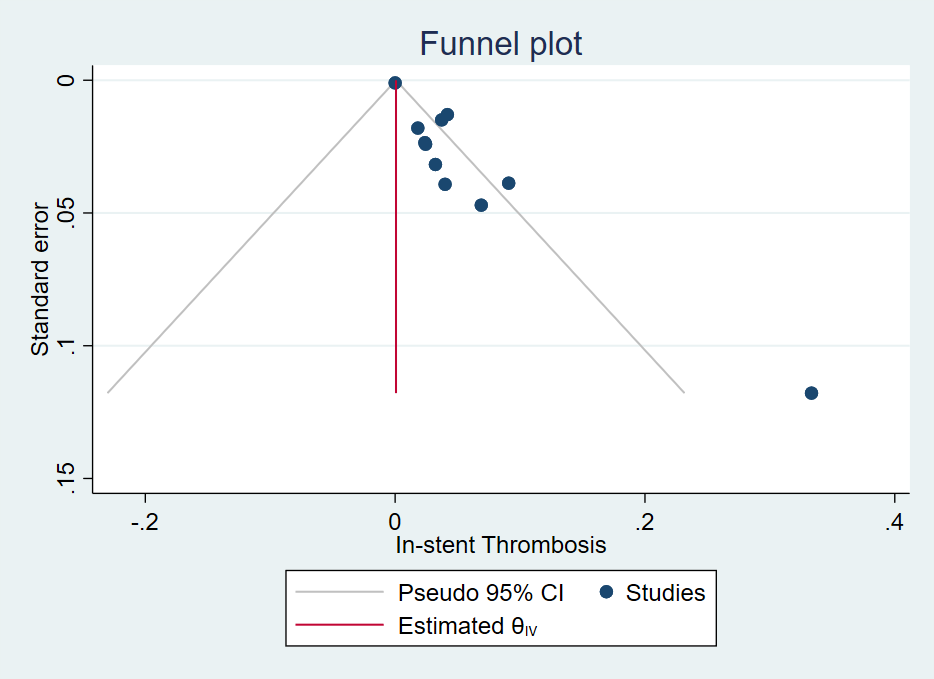


Figure 34 In-stent thrombosis publication bias


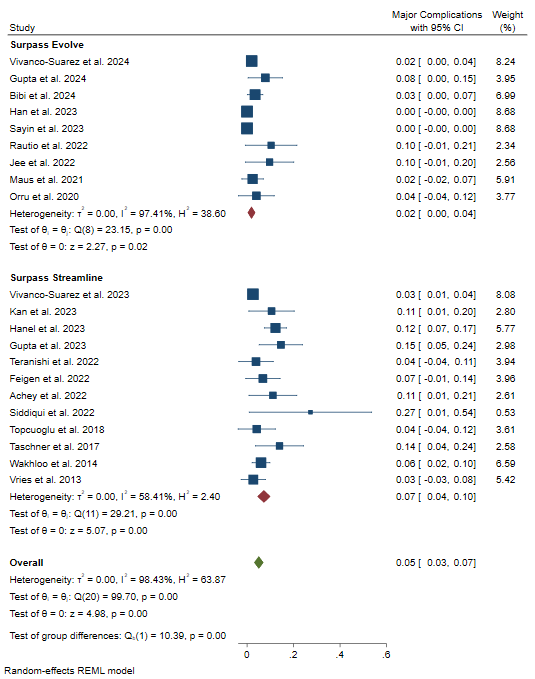


Figure 35 Major complications


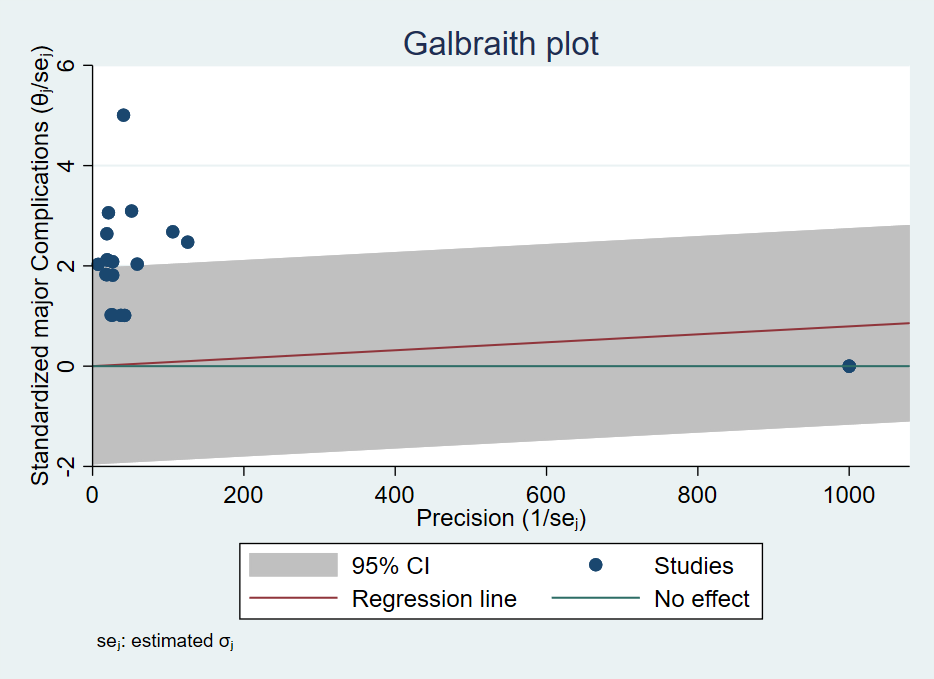


Figure 36 Major complications heterogeneity


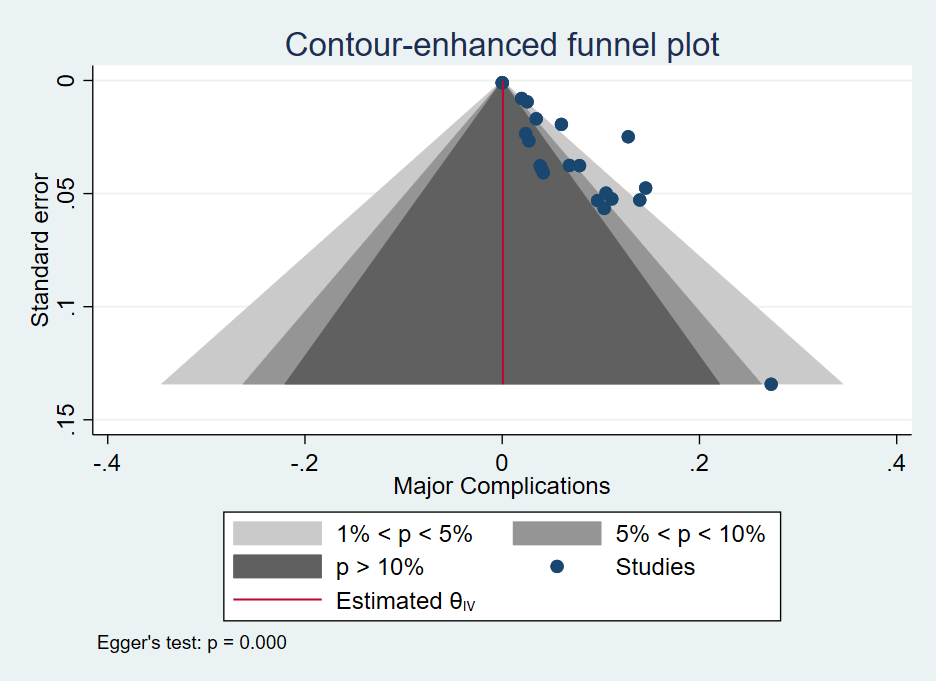


Figure 37 Major complications publication bias


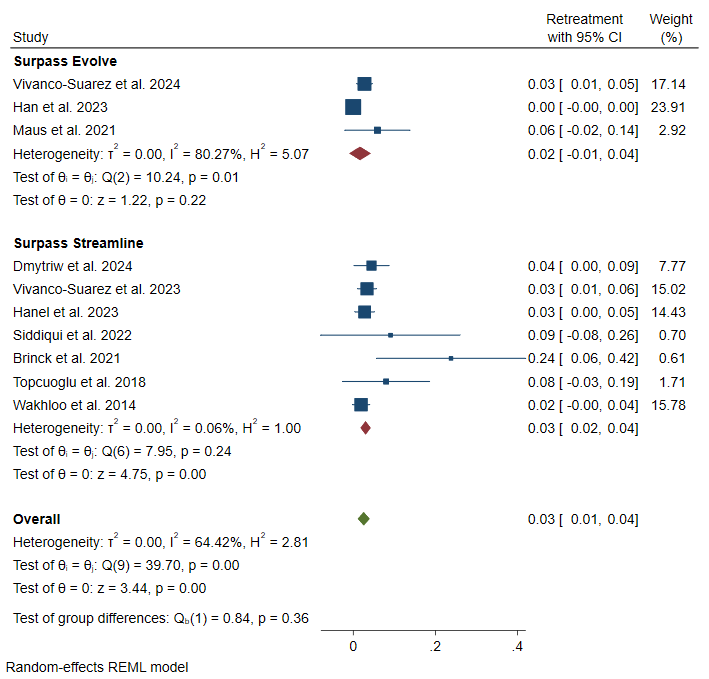


Figure 38 Retreatment


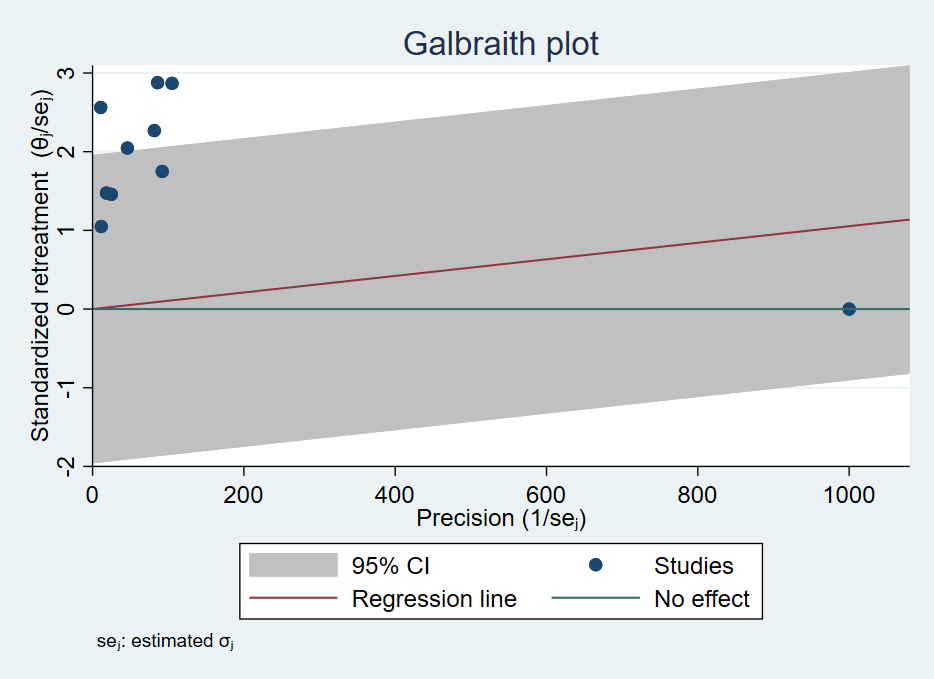


Figure 39 Retreatment heterogeneity


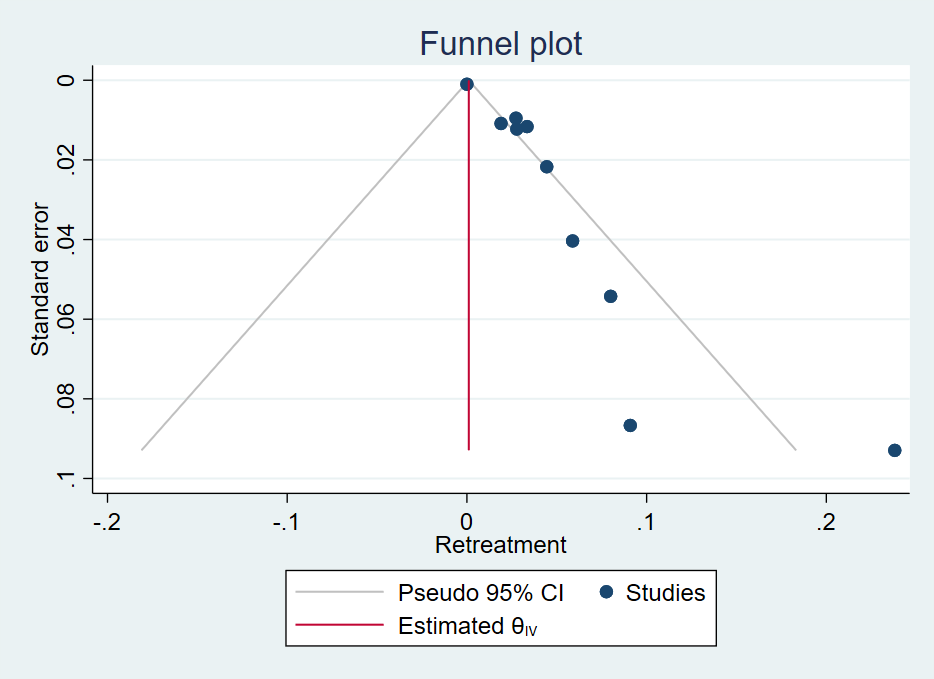


Figure 40 Retreatment publication bias


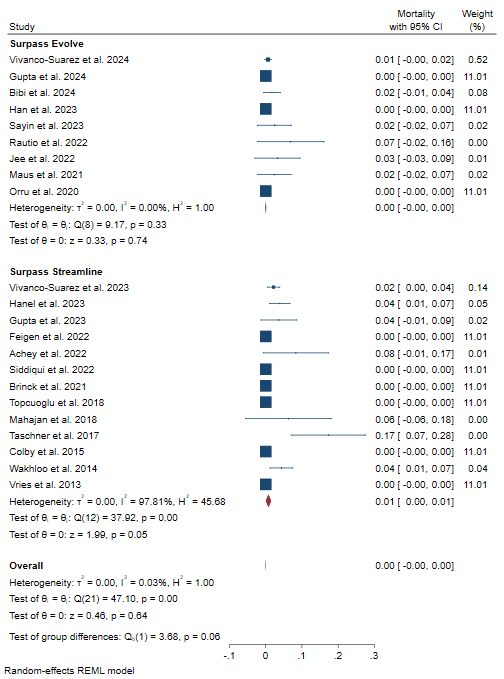


Figure 41 Mortality


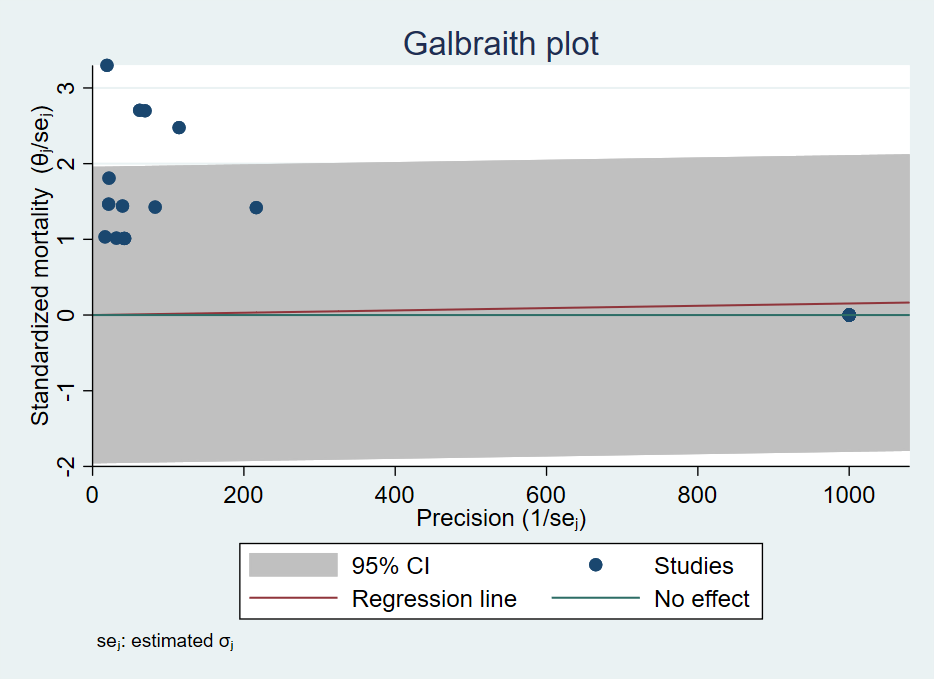


Figure 42 Mortality heterogeneity


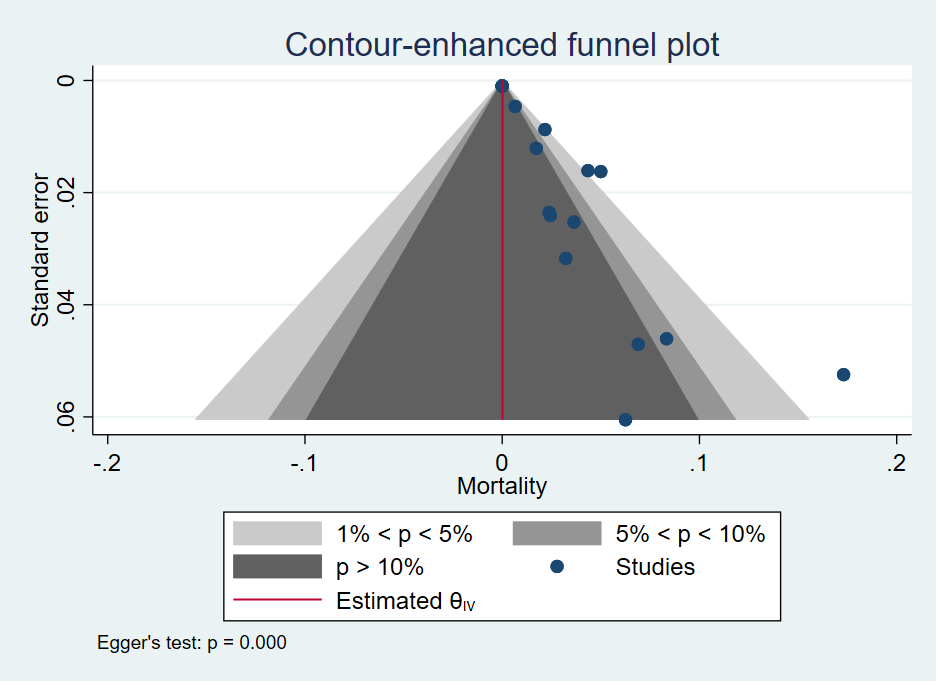


Figure 43 Mortality publication bias


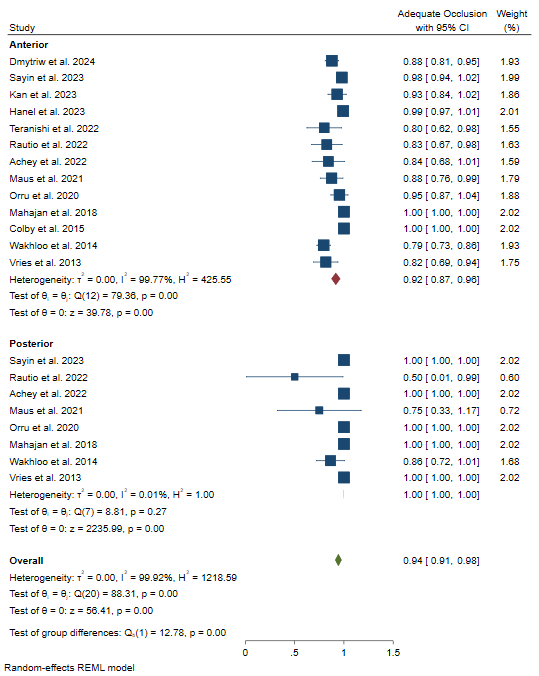


Figure 44 Adequate occlusion subgroup analysis on aneurysm location


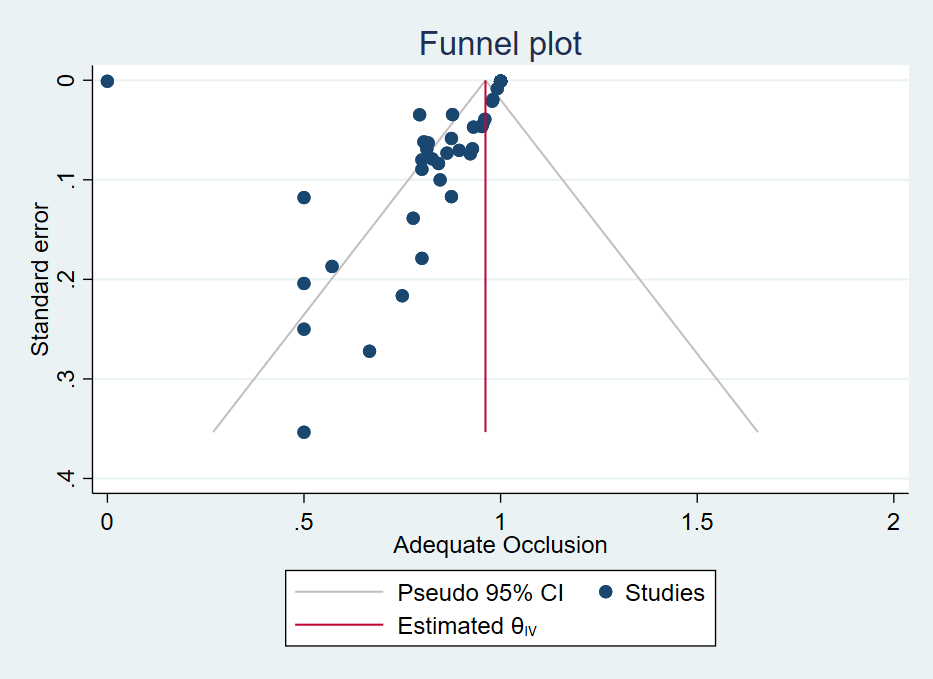


Figure 45 Adequate occlusion subgroup analysis on aneurysm location publication bias


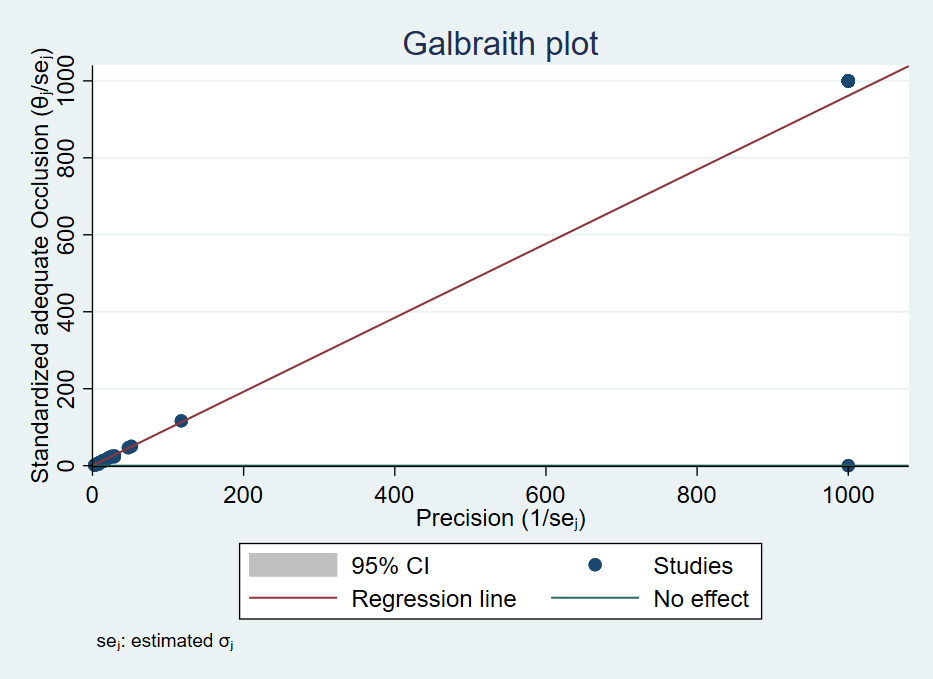


Figure 46 Adequate occlusion subgroup analysis on aneurysm location heterogeneity


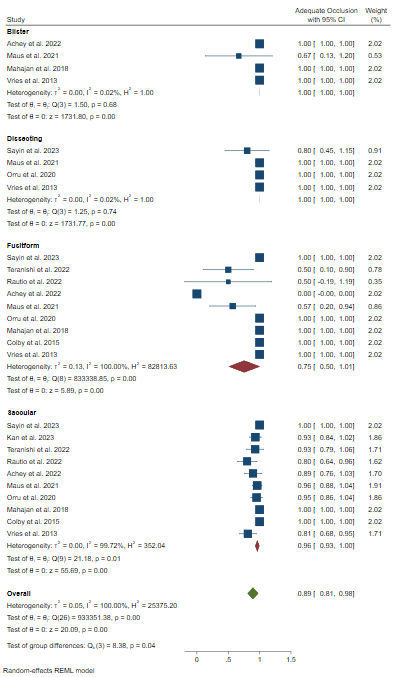


Figure 47 Adequate occlusion subgroup analysis on aneurysm morphology


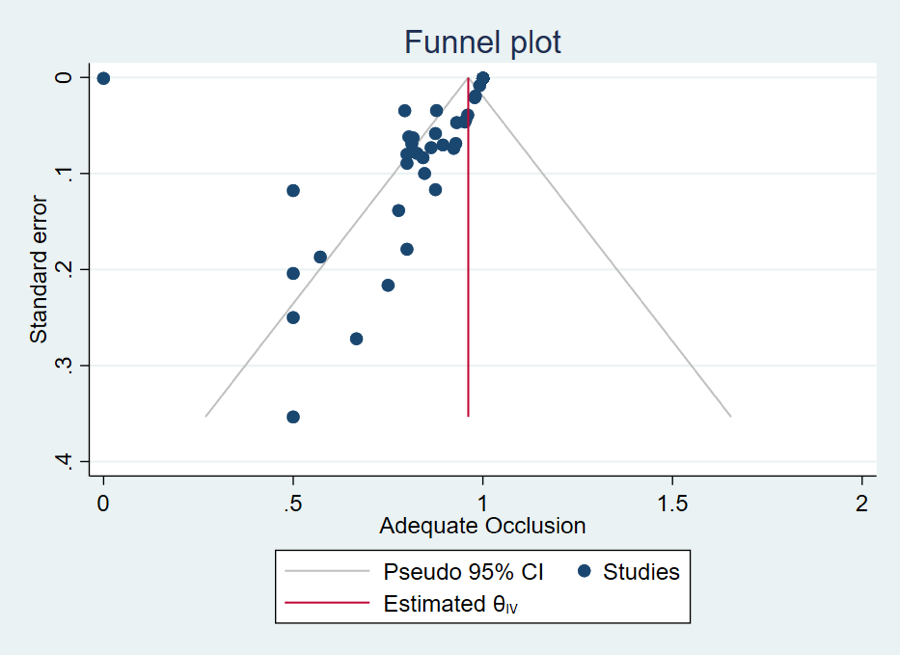


Figure 48 Adequate occlusion subgroup analysis on aneurysm morphology publication bias


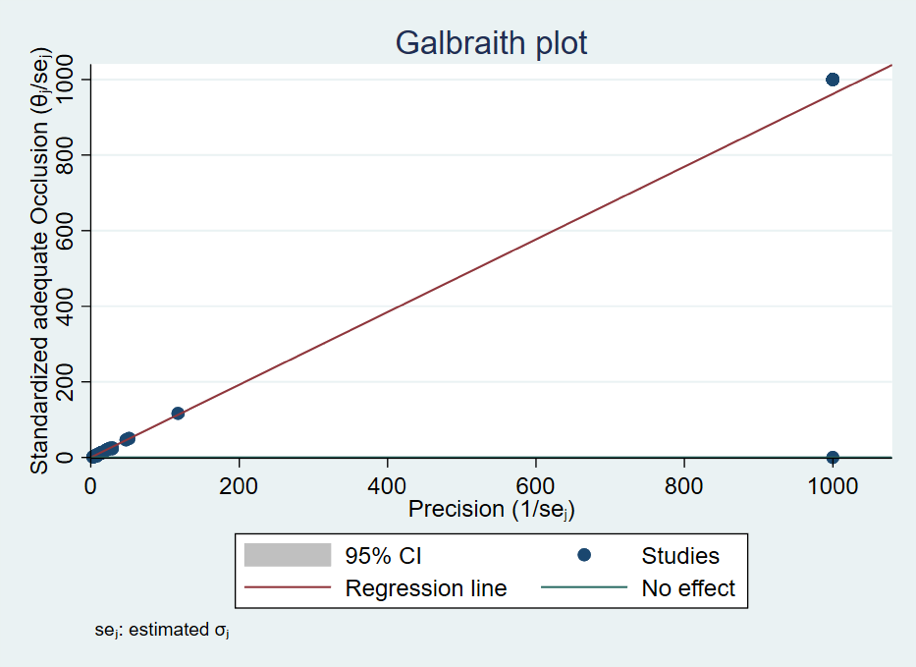


Figure 49 Adequate occlusion subgroup analysis on aneurysm morphology heterogeneity


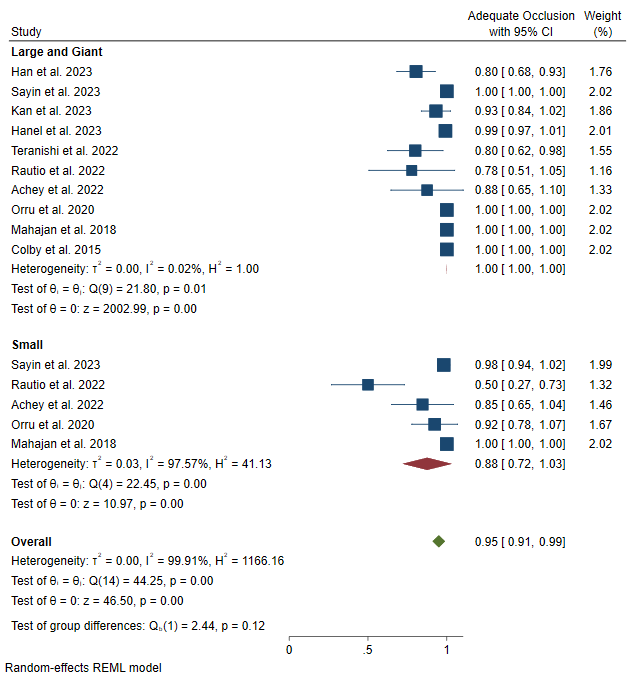


Figure 50 Adequate occlusion subgroup analysis on aneurysm size


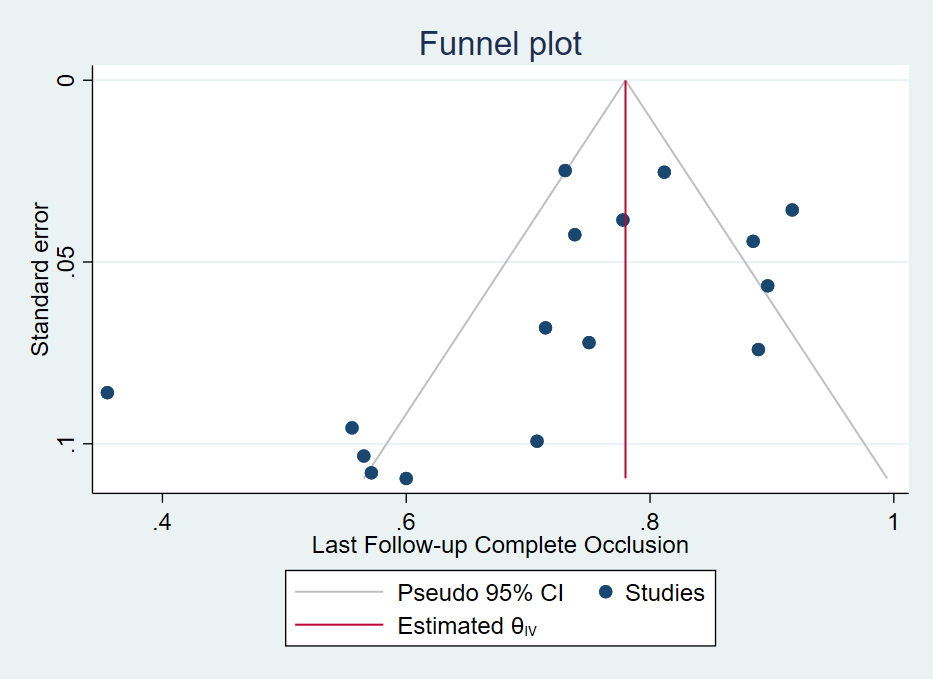


Figure 51 Adequate occlusion subgroup analysis on aneurysm size publication bias


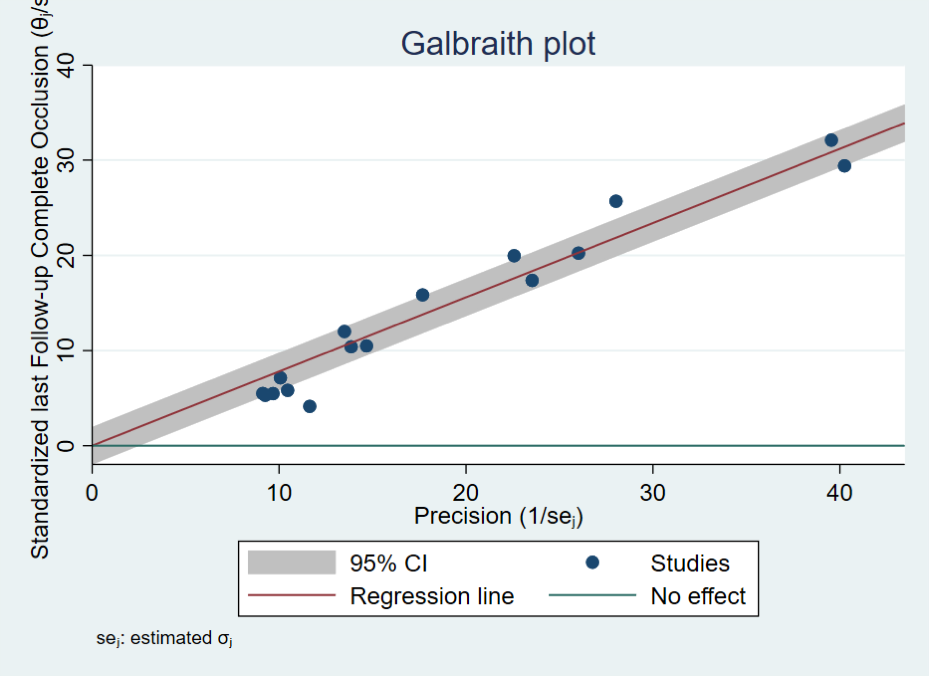


Figure 52 Adequate occlusion subgroup analysis on aneurysm size heterogeneity


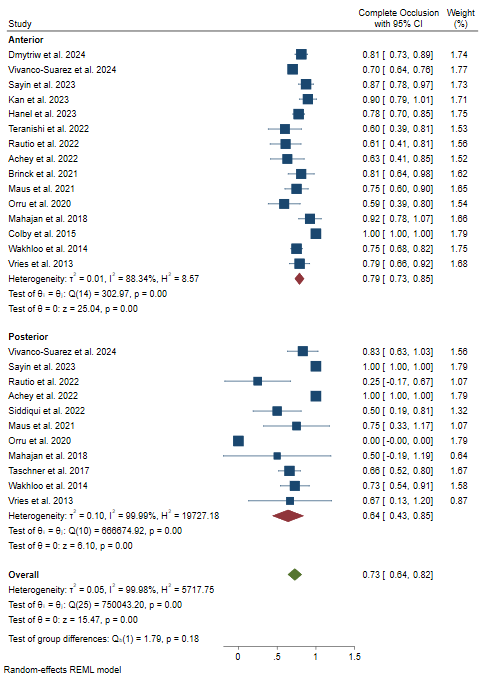


Figure 53 Complete occlusion subgroup analysis on aneurysm location


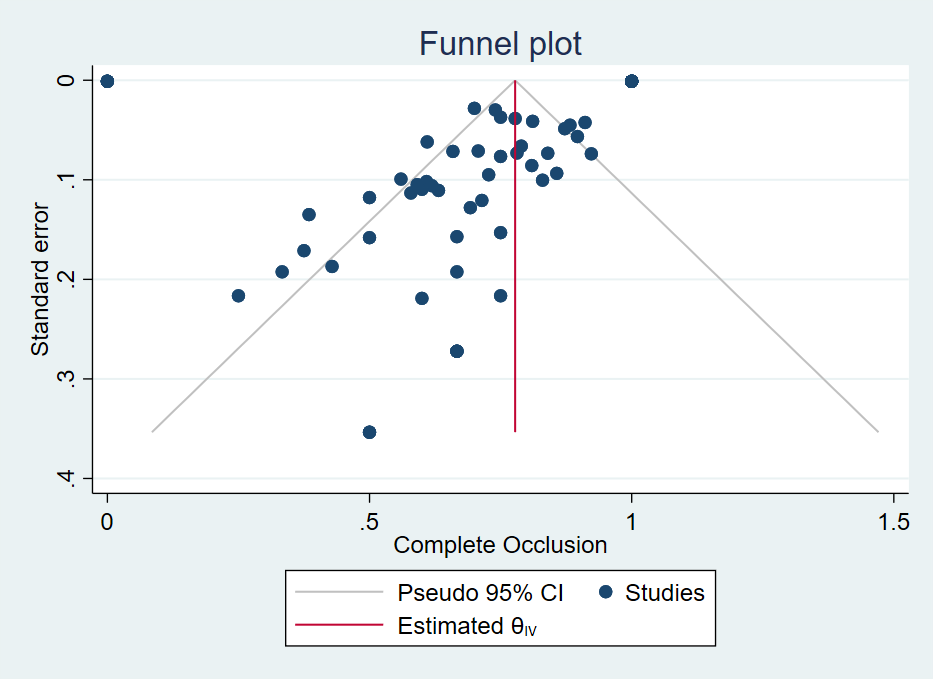


Figure 54 Complete occlusion subgroup analysis on aneurysm location publication bias


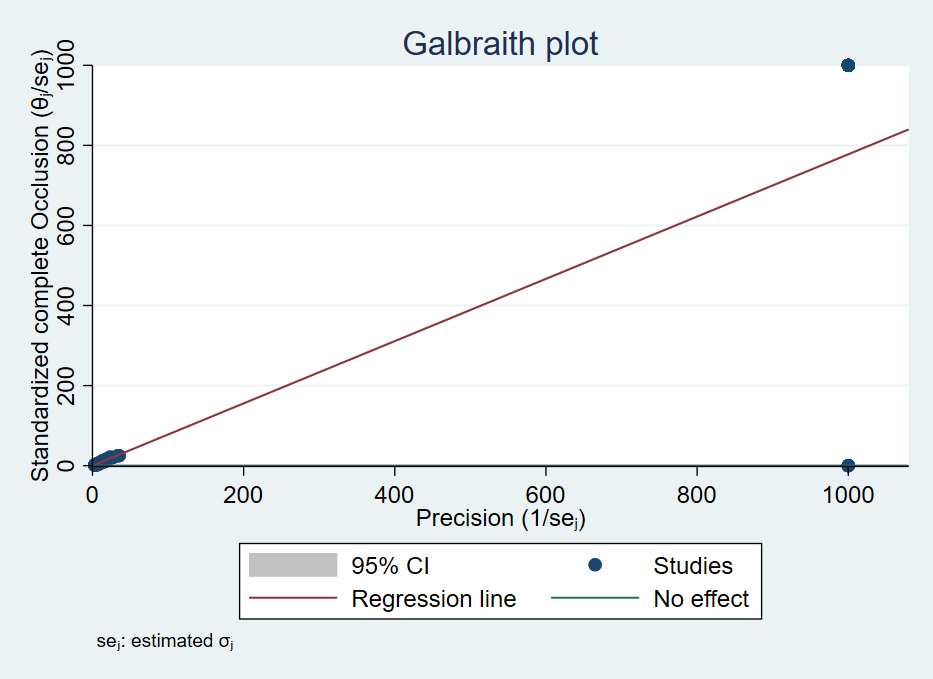


Figure 55 Complete occlusion subgroup analysis on aneurysm location heterogeneity


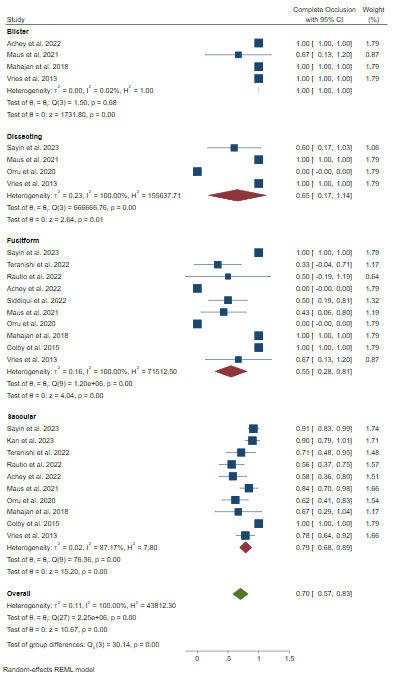


Figure 56 Complete occlusion subgroup analysis on aneurysm morphology


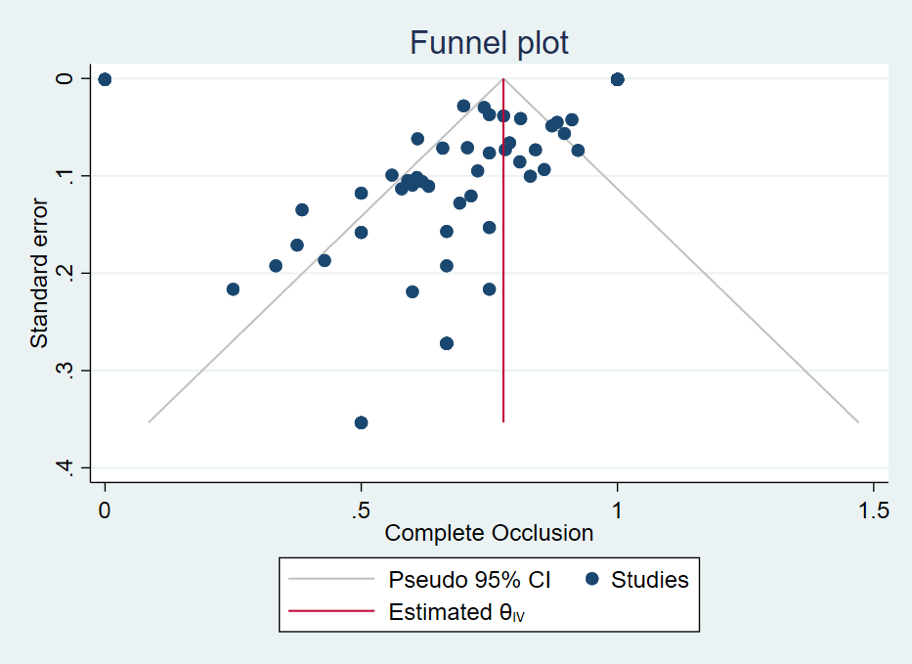


Figure 57 Complete occlusion subgroup analysis on aneurysm location publication bias


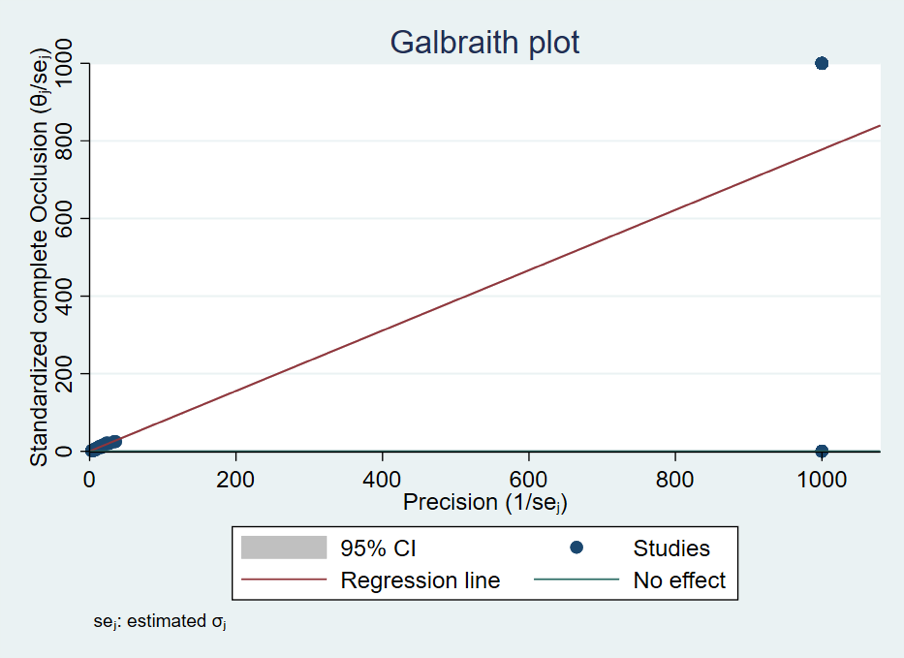


Figure 58 Complete occlusion subgroup analysis on aneurysm location heterogeneity


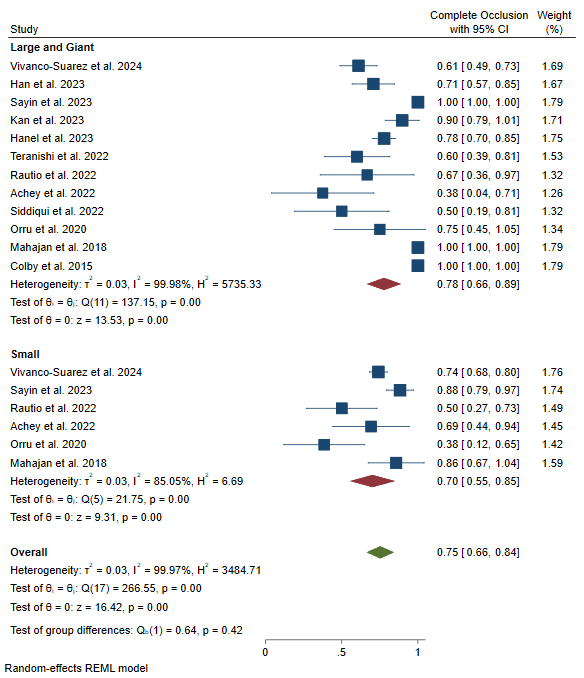


Figure 59 Complete occlusion subgroup analysis on aneurysm size


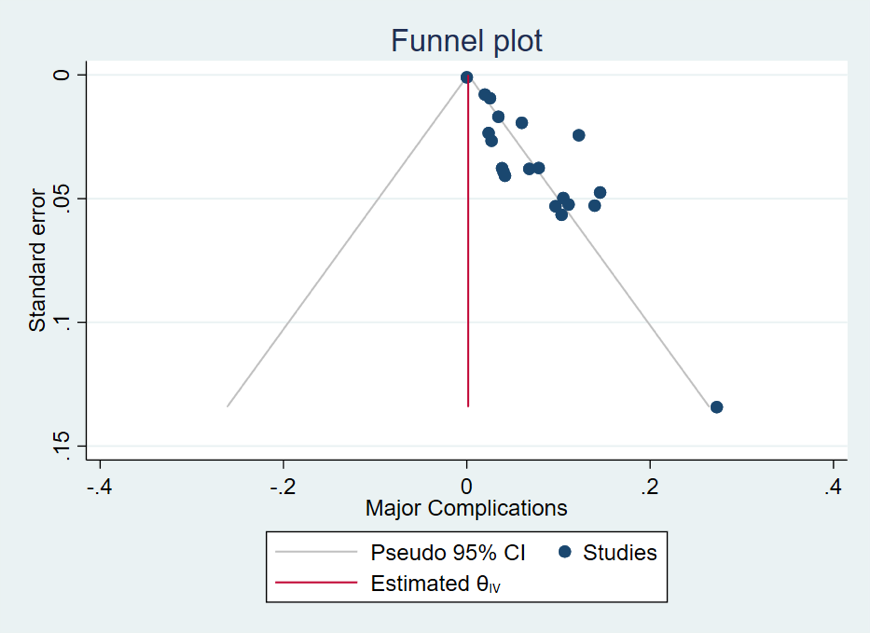


Figure 60 Complete occlusion subgroup analysis on aneurysm size publication bias


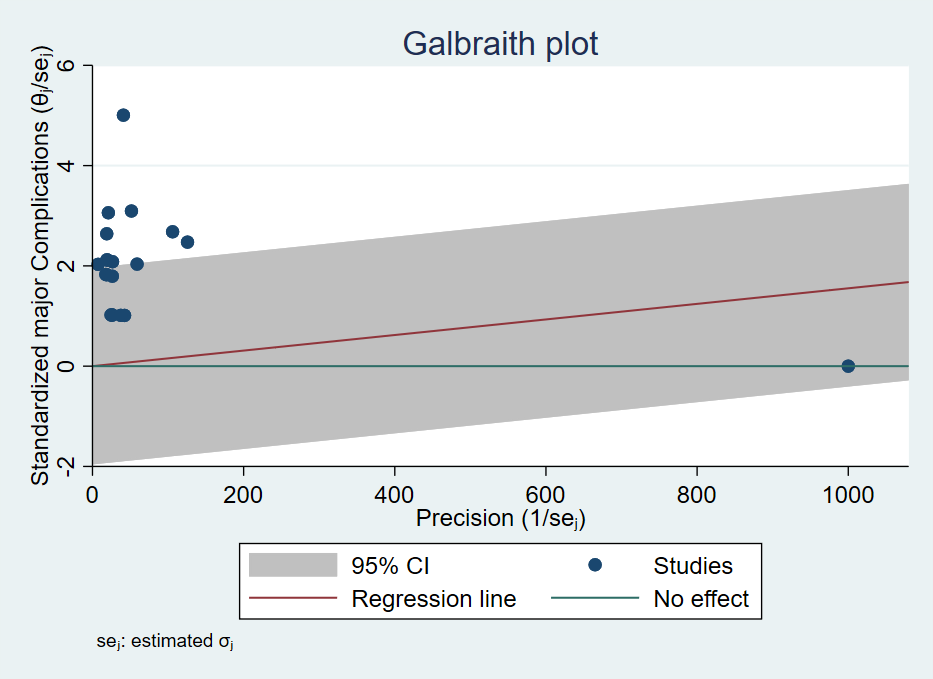


Figure 61 Complete occlusion subgroup analysis on aneurysm size heterogeneity
